# Multi-omics assessment of dilated cardiomyopathy using non-negative matrix factorization

**DOI:** 10.1371/journal.pone.0272093

**Published:** 2022-08-18

**Authors:** Rewati Tappu, Jan Haas, David H. Lehmann, Farbod Sedaghat-Hamedani, Elham Kayvanpour, Andreas Keller, Hugo A. Katus, Norbert Frey, Benjamin Meder

**Affiliations:** 1 Institute for Cardiomyopathies Heidelberg (ICH), Heart Center Heidelberg, University of Heidelberg, Heidelberg, Germany; 2 DZHK (German Center for Cardiovascular Research), Partner Site Heidelberg/Mannheim, Mannheim, Germany; 3 Department of Genetics, Stanford University School of Medicine, Palo Alto, California, United States of America; 4 Department of Clinical Bioinformatics, Medical Faculty, Saarland University, Saarbrücken, Germany; University College London, UNITED KINGDOM

## Abstract

Dilated cardiomyopathy (DCM), a myocardial disease, is heterogeneous and often results in heart failure and sudden cardiac death. Unavailability of cardiac tissue has hindered the comprehensive exploration of gene regulatory networks and nodal players in DCM. In this study, we carried out integrated analysis of transcriptome and methylome data using non-negative matrix factorization from a cohort of DCM patients to uncover underlying latent factors and covarying features between whole-transcriptome and epigenome omics datasets from tissue biopsies of living patients. DNA methylation data from Infinium HM450 and mRNA Illumina sequencing of n = 33 DCM and n = 24 control probands were filtered, analyzed and used as input for matrix factorization using R *NMF* package. Mann-Whitney *U* test showed 4 out of 5 latent factors are significantly different between DCM and control probands (*P*<0.05). Characterization of top 10% features driving each latent factor showed a significant enrichment of biological processes known to be involved in DCM pathogenesis, including immune response (*P* = 3.97E-21), nucleic acid binding (*P* = 1.42E-18), extracellular matrix (*P* = 9.23E-14) and myofibrillar structure (*P* = 8.46E-12). Correlation network analysis revealed interaction of important sarcomeric genes like Nebulin, Tropomyosin alpha-3 and ERC-protein 2 with CpG methylation of ATPase Phospholipid Transporting 11A0, Solute Carrier Family 12 Member 7 and Leucine Rich Repeat Containing 14B, all with significant *P* values associated with correlation coefficients >0.7. Using matrix factorization, multi-omics data derived from human tissue samples can be integrated and novel interactions can be identified. Hypothesis generating nature of such analysis could help to better understand the pathophysiology of complex traits such as DCM.

## Introduction

Dilated cardiomyopathy (DCM), which affects a considerable fraction of the global population (estimated prevalence– 1:250 [1]), is a structural disease of the heart caused by a dilated left-ventricle leading to a reduced ejection fraction in the absence of coronary artery disease or other heart conditions. The disease has a strong genetic component, with known causal mutations in more than 20–40 genes, which code proteins that are mostly part of the contractile fiber and its anchoring of the cardiomyocyte [2–4]. An altered transcriptional and epigenomic landscape was previously observed in patients with DCM, showing that methylation of DNA serves as an important regulatory mechanism of myocardial gene expression [5, 6] in the context of heart failure and/or DCM [7, 8]. Using whole-genome approaches, our group recently showed the strong interaction of gene expression and DNA methylation not only in peripheral blood but also myocardium of DCM patients [9].

**Fig 1:**
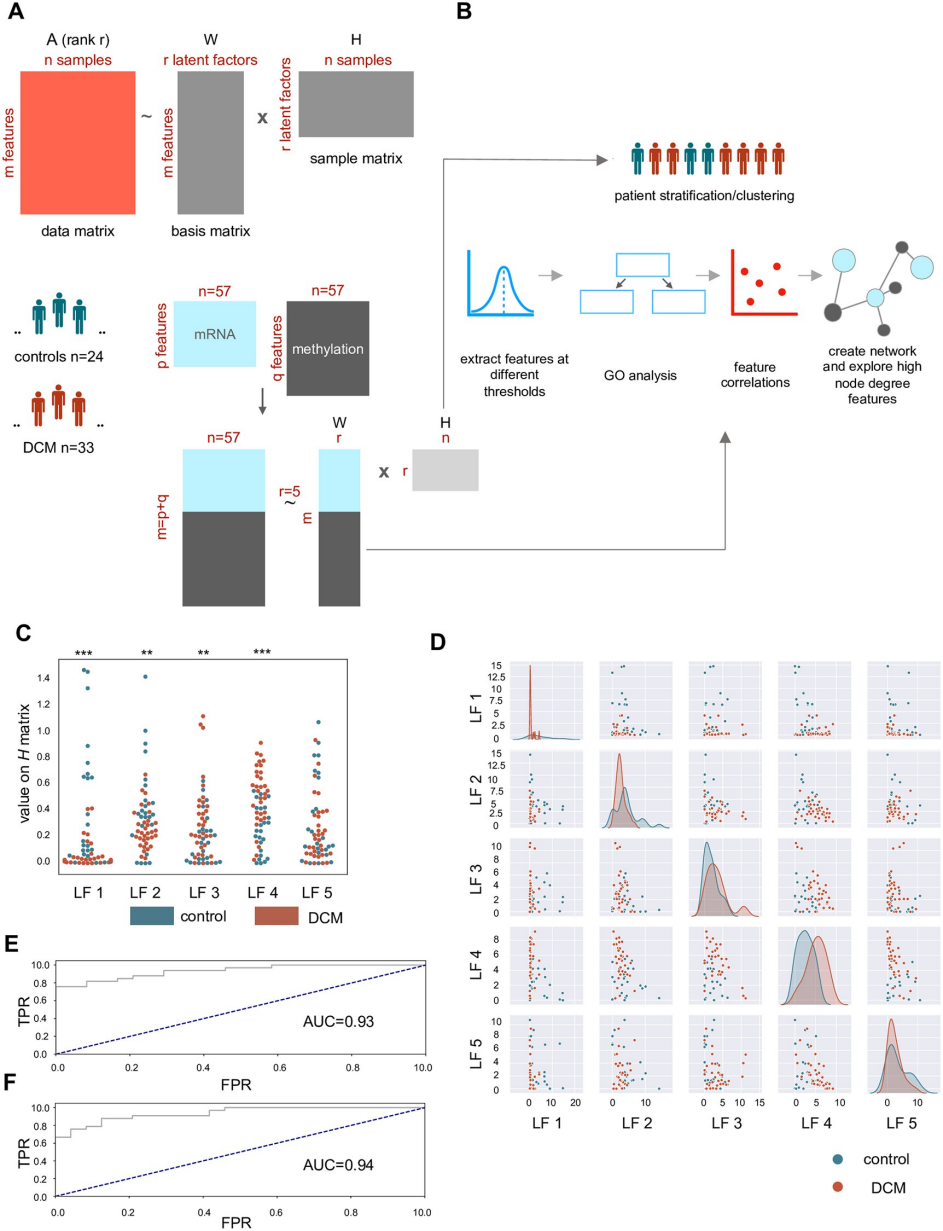
Overview of matrix factorization and distribution of latent factors. **A)** The general concept of matrix factorization is detailed in the figure. Matrix A with *m* features and *n* samples can be decomposed at rank *r* in two matrices, one signifying the relationship of the original features with the latent factors (*W* matrix) and another signifying the relationship of the latent factors and the samples (*H* matrix). The cohort with 24 control and 33 DCM samples is profiled for gene expression and methylation and matrix factorization is carried out on the combined methylation and gene expression data matrices at rank 5. **B)** The *H* matrix is used for clustering of patients and determining the potential of the latent factors in discriminating DCM from control samples. From the *W* matrix, features with high loadings above a threshold (90^th^ percentile) are selected and GO analysis is performed, followed by correlations and network analysis. **C**) Swarm plot of latent factor values. *** = *P*<0.0005, ** = *P*<0.005 as per a Mann-Whitney *U* test. **D**) Scatterplots of the latent factors show the pairwise distribution of latent factor values for DCM and control samples. **E**) Receiver operating characteristic curve for differentiating between DCM and control with the 5 latent factors. TPR = true positive rate. FPR = false positive rate. **F**) Receiver operating characteristic curve using 4 significant latent factors.

A combined analysis of gene expression and methylation data is thought to be beneficial for understanding the interactions between molecular layers, enabling definition of quantitative trait loci [10]. Several newer statistical techniques are of potential interest for such integration of bi- or multi-omics datasets. Notable amongst them are unsupervised dimension reduction methods like non-negative matrix factorization (NMF), principal component analysis and multiple-factor analysis [11]. NMF decomposes an original matrix into its lower dimensional representation. The decomposition is additive, meaning that it explains the original data matrix as a linear combination of its parts. More specifically, given a large dataset with multiple samples and large number of measured variables, matrix factorization will condense the variables into a smaller number of variables (equal to the factorization rank), with each original variable making a contribution of a certain magnitude to a so-called latent variable. It produces two matrices; one corresponds to the amplitude with which each feature in the original matrix contributes to the latent factor and is called the basis matrix. The other matrix relates the weight of the samples on the latent factors and is called the sample/encoding matrix [12–16]. The identified latent factors thus are representative of the underlying major pathways/processes contributing to most variation in the dataset. Several flavors or algorithms of NMF exist. Using NMF is of great advantage as it reduces the large number of variables that multi-omics datasets produce, into easily interpretable latent factors shared across the datasets. Fig 1A is a schematic representation of the concept of NMF.

The first applications of non-negative matrix factorization were in the field of image analysis and facial recognition [17, 18] and subsequently several data intensive fields used NMF or its variations. NMF also finds applications in the field of medicine where, for example, it has been used to analyze PET images [19]. In cardiology, NMF has been used for diagnostics and risk assessment [20, 21]. In bioinformatics and molecular biology, it is applied on RNA-seq and single cell RNA-seq data with the aim of finding ‘metagenes’, which are groups of co-regulated individual genes [22, 23]. It has been used to cluster cancer subtypes, and to detect protein motifs [24, 25]. Several multi-omics studies use NMF for integrated analysis of molecular data types [26–34]. Glezeva et al. [35] have used NMF for clustering of a cohort of DCM samples and controls using a methylation profile generated using BIS-seq. So far, to the best of our knowledge, NMF has not been used for an integrated analysis of gene expression and methylation data types from human cardiac tissue or more specifically a DCM cohort.

In this study, we integrated transcriptome and methylome data of a cohort of DCM patients using NMF (S1 Table). We start by decomposition of the concatenated transcriptome and methylome matrix into latent factors and extract features for each latent factor that fall within the 90^th^ percentile of coefficient on *W* matrix. From these top-contributing features, we assess if they represent specific biological pathways. We then use the gene and CpG features obtained per latent factor for performing pairwise correlations using Pearson’s coefficient. This is followed by creation of networks to help identify important hubs and genes connected with CpG features. Whereas most studies have used NMF mainly for sample clustering, here we carry out a deeper exploration of the basis matrix (*W* matrix), which allowed us to find correlated features and hub genes. We validate the results in an independent patient cohort (S2 Table) and compare the results to an m-QTL analysis. Fig 1B shows the subsequent statistical analysis carried out on the NMF matrices.

## Materials and methods

### DCM and control cohort data

The cohort recruitment was approved by the ethics committee and medical faculty of Heidelberg University. All participants have given informed consent to allow the molecular characterization of their biopsy samples. The consent was given in writing. Detailed information on patient enrollment and assessment of relevant clinical parameters for both discovery and validation cohort has been provided in previous publications [9, 36]. S1 Table provides details on the discovery cohort and S2 Table describes the validation cohort.

### RNA sequencing data analysis

RNA sequencing using poly(A) enrichment of the mRNA was performed for a total of 57 samples, comprising of 33 DCM and 24 controls. A median read depth of 36.72 million was achieved per pair-ended sequencing data, with a read length of 75 base pair. The reads were subjected to a quality check using the tool FastQC [37]. The reads were then aligned to human genome GRCh38.p12 using the tool Hisat2 (2.1.0) [38] using the splice-aware option The parameters used were “hisat2 -p 8—dta -x grch38.p12–1 input.r1–2 input.r2 > output.sam”. SAM files thus obtained were converted to BAM files and sorted using samtools. BAM files were used as an input to the featureCounts [39] tool of the Subread package, to count the number of reads aligning to the gene features as defined in the GTF file Homo.sapiens.GRCh38.98.gtf obtained from Ensemble. For the counting, only uniquely mapping fragments were considered. S1A Fig shows the total number of fragments (pair of forward and reverse reads) and the assignment rate.

Read counts were imported in R package *DESeq2* [40], for normalization of sequencing depth and analysis of the differentially expressed genes between DCM and control. The normalized counts for a total of 58,303 genes is visualized as an MA plot and as a volcano plot. Further, *rlog* was used for transformation of the normalized count data. The basic statistics of the gene expression matrix are detailed in S3 Table. Before using this matrix as an input for matrix factorization, the genes were filtered as per their mean values and variance across samples. Additionally, PCA was performed to visualize the presence of obvious batch effects, but no correction was required. Unsupervised clustering by the k-means algorithm was performed using the Python library *scikit-learn* to determine relationships between samples. This was done using the first 5 principal components (PC).

### Illumina methylation 450K array data analysis

The Infinium 450K array was used for probing methylation sites and was analyzed as described in detail in the previous publication [9]. Important steps included quality check, normalization; the correction for batch effects and correction for principal components 1–4, age and gender. The beta-value matrix was imported in the R package *limma*. Beta-values were converted to M-values and differentially methylated regions were calculated. The differentially methylated regions were visualized as a volcano plot. Beta matrix with probes passing the initial quality check (consisting of CpG and non-CpG methylation) was used to carry out mean and variance filtering to select for highly methylated variant CpG sites. A summary of the methylation data matrix is provided in the S4 Table.

### Non-negative matrix factorization

The mean and variance filtered gene expression and methylation array data matrices were used as an input for carrying out a joint non-negative matrix factorization. Specifically, the samples that had both gene expression and methylation data modalities profiled for them were selected and the matrices were concatenated. This was followed by a normalization carried out using *scikit-learn’s normalize* function using the ‘max’ norm. The normalized concatenated matrix was used first to determine the factorization rank.

The R package *NMF* was used for performing the factorizations [41]. To find an optimal factorization rank, first we ran the algorithm for 99 iterations, from rank 2 to rank 100, increasing the rank by 1 in each iteration. For each iteration we calculated the residual sum of squares (RSS), explained variance and delta explained variance. We inspected the delta explained variance values and selected *r* = 5, since the explained variance sees a sharp drop after *r* = 5, with some increase again at rank 7 and 8. We then build models using rank 2, 3, 4 and 5 using a seed of 111223 for reproducible results and total of 30 runs for obtaining a stable result. We also ran the algorithm 3 times for rank = 5 using different seeds. A summary of the matrix factorizations performed is summed up in S1 File.

### Downstream analysis of latent factor profile

The downstream analysis of the matrix factorization comprised of two main kinds of analyses: the analysis of the latent factor profile (*H* matrix) and the analysis of the amplitude matrix (*W* matrix). For each latent factor a Mann-Whitney *U* test was performed to test the difference in distribution between the DCM and control samples. Multivariate logistic regression was carried out to test the predictive value of the five latent factors. The latent factor profile was then used for an unsupervised clustering of the samples using the k-means algorithm from *scikit-learn*. K-means was performed for *k* = 4:7 and information gain was calculated using the equation:

$$E=-\sum_{i}^{C}p_{i}{log}_{2}p_{i}.$$

A Chi square test was used to detect the significance of the clustering under the null hypothesis that the clusters do not separate DCM and control. The *P* value so obtained was also compared with values obtained for k-means clustering performed for RNA-seq and methylation data matrices. Latent factors obtained from all four models from rank 2:5 were also correlated.

### Downstream analysis of feature matrix

For each latent factor in the model with rank = 5, the distribution of the weights of the features was visualized. All the features having a weight within the 90^th^ percentile (top 10%) were further selected and a GO term analysis was performed with R package *goseq* [42]. The significant GO terms were selected based on FDR corrected *P* values. Pearson correlations with FDR adjusted *P* values were produced for top 10% selected gene and CpG features. The correlations were used to create a network using Python’s *Networkx* library. Node degree for each feature was calculated. The gene and CpG associations found in the discovery cohort were tested in the separate validation cohort.

### Comparison with m-QTL analysis

The genes part of significant correlations were considered for an methylation-expression QTL analysis. Specifically, for each gene that was considered, all CpG sites present on the same chromosome 10,000 base-pairs upstream or downstream were selected for the QTL calculations. Correlating pairs were visualized as a Circos plot. All analysis was performed using custom Python and R scripts which are available at https://github.com/rewatitappu/NMF_analysis_toolkit. Summarized counts of the RNA-seq and methylation data are available at https://ccb-web.cs.uni-saarland.de/cms. Additional supplementary material/data is also available on request.

## Results

### Gene expression and CpG methylation profiles are characteristic for DCM

From a prospective DCM cohort of 135 patients, those who had high quality RNA-seq (poly(A) enriched) and methylation data (Illumina HM450) from the myocardium were included in data analysis. Patients free of heart failure or DCM who had undergone heart transplantation were used as controls. Details on data generation and clinical characteristics are described in [9]. The final multi-omics dataset comprised 57 samples, with 24 controls and 33 DCM patients (Fig 1A, Table 1, S1 Table).

**Table 1:** An overview of the discovery cohort.

	Age at visit	BMI	NYHA	Gender
DCM	53.39	26.88	2.25	25 males, 8 females
Controls	53.31	24.42	1.29	20 males, 4 females

The table provides an overview of the entire cohort (57 samples). The body mass index (BMI) and NYHA (New York Heart Association) parameters are high for DCM as compared to controls.

First, we performed differential gene expression and differential methylation analysis of DCM and control and performed unsupervised hierarchical clustering of samples (S1–S4 Figs). In order to confirm whether the gene expression is indicative of a DCM phenotype, we checked the expression of known heart failure markers like *NPPA* and *NPPB*, and the expression of these genes were indeed 1.7-fold (*P =* 0.01) and 1.6-fold (*P =* 0.02) higher in DCM as compared to controls, respectively. Gene ontology (GO) analyses for the genes up-regulated in DCM (corrected *P*<0.05) as compared to controls resulted in GO terms related to cellular components of extracellular space (*P* = 5.05E-3) and transmembrane transporter complex (*P* = 3.22E-03); molecular function of receptor ligand activity (*P* = 1.6E-6) and biological process of regulation of ion transport (*P* = 2.45E-1). GO analysis of DCM up-regulated CpG sites resulted in biological adhesion (*P* = 3.69E-07), homophilic cell adhesion via plasma membrane adhesion and cell adhesion (*P* = 6.9E-07) as the main processes.

### Latent factor profile offers a means of stratification of DCM and control samples

**Fig 2:**
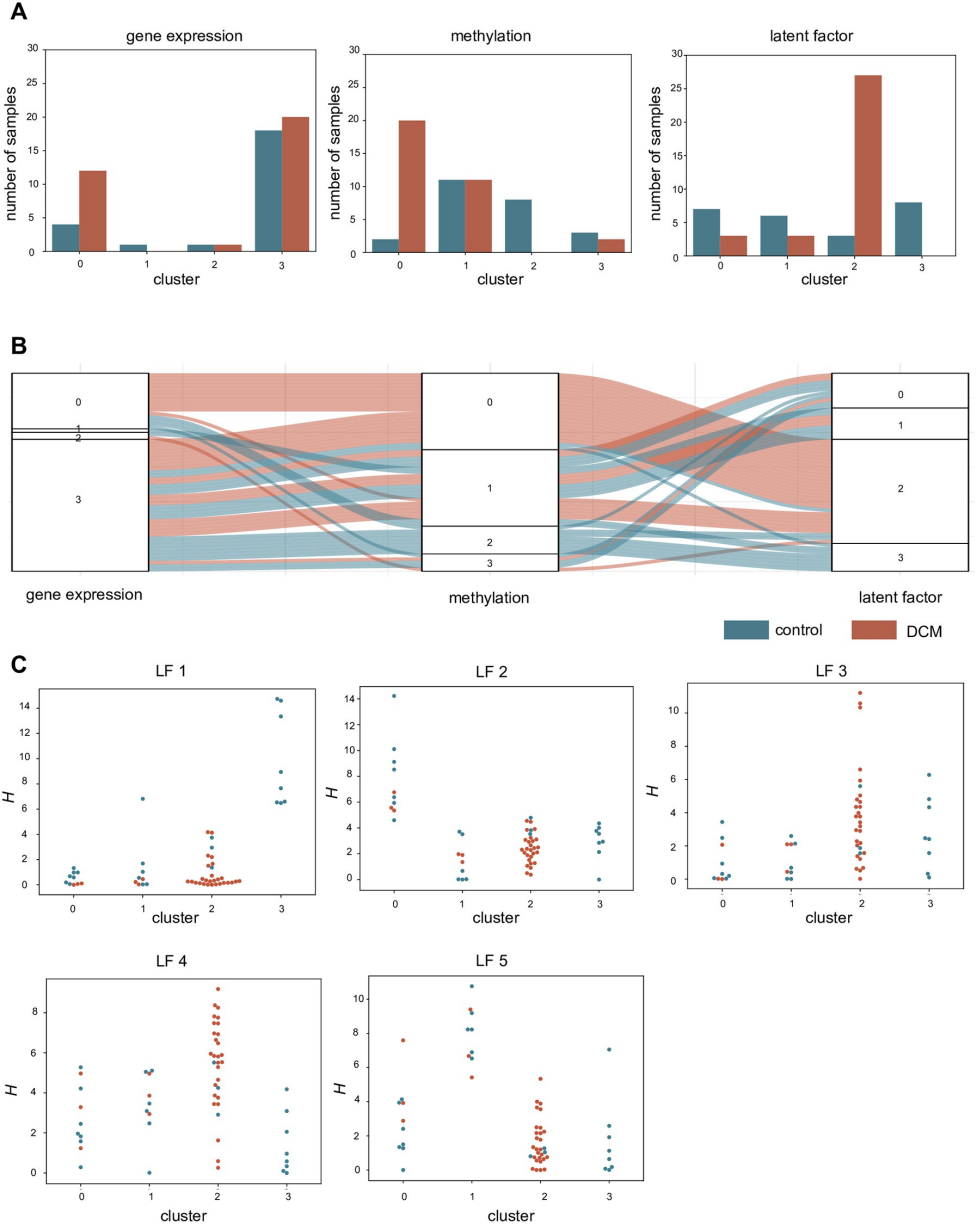
Clustering of the samples with latent factor profile. **A)** Clusters obtained using the *k-means* algorithm at *k* = 4 for the gene expression data matrix, methylation data matrix and latent factor profile. Clusters 0, 1, 2 and 3 are plotted on the X-axis and Y-axis represents the number of control and DCM samples in each cluster. **B)** A Sankey-flow diagram depicts the flow of samples between the 4 clusters as per the gene expression, methylation and latent factor profile. We see that as per the latent factor profile, several DCM samples (27) are binned into cluster 2. **C)** Swarm-plots depicting the value of five latent factors for samples in each cluster as obtained by the latent factor profile show which samples have an over-expression of that particular latent factor. We see that for cluster 2 in which DCM samples predominate, have a high value for latent factors 3 and 4.

Concatenated mean and variance filtered RNA-seq and methylation data matrices (Table 2) were used for performing NMF at rank 5 (Fig 1C and 1D). Out of the 5 latent factors obtained; 1, 2, 3 and 4 are significantly differentially distributed (*P*<0.05) between DCM and control samples as per a Mann-Whitney *U* test (Table 3). Similar results were obtained with a logistic regression analysis, where latent factors 1–4 have *P*<0.05 and model using all 5 latent factors returns an area under the curve (AUC) of 0.93 whereas a model using latent factor 1–4 yields AUC of 0.94 (Fig 1E and 1F). K-means clustering (*k* = 4) retrieves a cluster with 27 DCM samples and 3 controls samples and another cluster with 8 controls (Fig 2A). The other two clusters are more heterogeneous, with both DCM and control samples. K-means using only 5 latent variables derived from joint analysis of mRNA and methylation features was compared to k-means from individual RNA-seq and methylation data matrices. For *k* = 4,5,6 and 7, the information gain evaluated revealed that latent factor profile had the highest average information gain, at 0.44, while values for mRNA and methylation profiles were 0.08 and 0.29, respectively (S5 Table). Thus, the latent factors which are a condensation of the combination of methylation and mRNA features contribute to a better separation of DCM and control samples as compared to only individual matrices of methylation and RNA-seq data. When we considered only top 1000 variable genes in the RNA-seq data, the information gain was higher than using first 5 PC, at 0.57. However, for a fair comparison with 5 latent factors, we compare it with 5 PCs. Through the sample matrix *H*, we inspected which patients have high values for a particular latent factor. Fig 2B and 2C shows the flow and distribution of latent factor values in the sample sets (DCM and controls).

**Table 2:** Number of gene (RNA-seq) and CpG (methylation array) features before and after mean and variance filtering.

	Mean	Variance	#Features	#Features after filtering
RNA-seq	0.02	0.01	58303	24026
methylation array	0.40	0.00097	394248	145411

Table provides the thresholds used for mean and variance filtering of the features to be used as input for matrix factorization and the total number of features selected as a result.

**Table 3:** Latent factors and threshold for further selection.

Latent Factor	Test statistic	*P*	*W* Threshold 90^th^ percentile	#Gene features	#CpG features
LF 1	164.0	9.14E-05	0.41	666 (220)	16280 (13138)
LF 2	264.0	0.01	0.62	1165 (32)	15781 (15359)
LF 3	270.0	0.02	0.52	576 (72)	16370 (13995)
LF 4	145.0	2.57E-05	0.86	42 (4)	16904 (16465)
LF 5	348.0	0.22	0.57	1031 (19)	15913 (13908)

The *P* values of the Mann-Whitney *U* test performed for testing the difference in distribution of the values of the latent variables across DCM and controls is given in the table. The *W* coefficient threshold as well as the number of gene and CpG features thus selected is summarized in the table. The number of features having a significant DCM association (*P*<0.05) is denoted in the brackets.

### Top ranked features per latent factor represent distinct biological pathways

**Fig 3:**
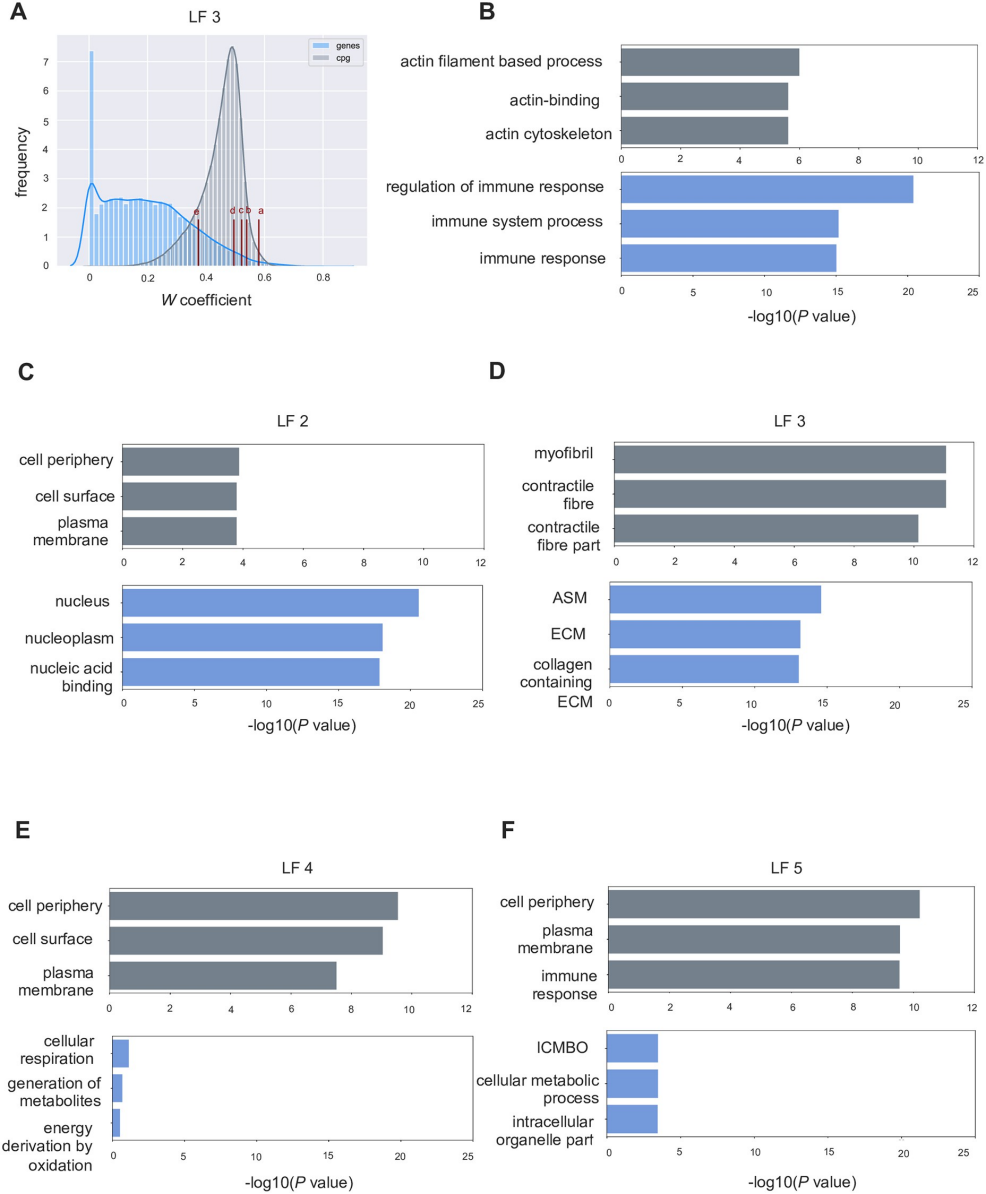
Distribution of *W* matrix coefficient and top gene ontology terms. **A)** The distribution plot of the *W* coefficients for gene and CpG features for latent factor 3. The labels a-e represent the 99^th^, 95^th^, 90^th^, 75^th^ and 25^th^ quantiles. **B-F)** Bar plots representing the log_10_ *P* value of significance of enrichment for a gene ontology term. For the selected features from each latent factor, (>90^th^ percentile), a GO terms analysis performed for gene and methylation features and the FDR corrected *P* for significance of the term is reported. **ASM** = anatomical structure morphogenesis, **ECM** = extra-cellular matrix, **ICMBO** = intracellular membrane-bounded organelle.

After using the sample matrix *H* (latent factor profile) for patient clustering, we set to characterize the latent factors in terms of their biological meaning. For this, we employed the coefficients per latent factor on *W* matrix and carried out gene ontology analysis using R package *goseq* for features selected at 5 thresholds– 25^th^, 75^th^, 90^th^, 95^th^ and 99^th^ percentiles (Fig 3A, Table 3). We report FDR corrected *P* values and consider *P*<0.05 as significant enrichment of a term. The pathways become more specific (lower in the GO hierarchy) as we go from lower thresholds to higher thresholds, as expected. For CpG features, threshold above the 90^th^ percentile did not yield significant pathways. We therefore selected top 10% features (90^th^ percentile) per latent factor. Disassembling the features showed that not all of them have a significant DCM association per se. On average 11.91% of the selected gene expression features per latent factor show a significant association with DCM, whereas an average of 89.46% of the selected CpG features per latent factor have a significant *P* of DCM association. Gene ontology analysis of selected gene features reveals that latent factor 1 represents mainly the biological process (BP) of immune response (*P* = 3.97E-21). Latent factor 2 represents the cellular component (CC) of nucleus (*P* = 2.70E-21) and genes selected for latent factor 3 show enrichment for GO term anatomical structure morphogenesis (*P* = 2.66E-15) and extracellular matrix organization (*P* = 7.09E-14). Latent factor 4 does not have any significantly enriched term at the desired threshold, however it tends to the biological process of cellular respiration (*P* = 0.07). Latent factor 5 represents the component of intracellular membrane-bound organelle (*P* = 0.003) amongst other processes. For CpG features, significant terms associated with latent factor 2, 4 and 5 are cell periphery (*P* = 2.88E-10) and plasma membrane (*P* = 9.24E-10). Latent factor 3 corresponded to myofibril and contractile fibril (*P* = 8.46E-12). We observe that latent factor 4 is strongly DCM associated, however, the top 10% features selected for this factor do not represent predefined GO terms, which deserves further attention. For latent factor 3, we see the highest percentage of the gene (mean for top 3 GO terms 11.66%) and CpG (mean for top 3 GO terms 60%) features enriched for the GO terms extracellular matrix and contractile fibril. Fig 3B–3F displays the top 3 GO terms associated with each latent factor.

To determine whether the selected 10% features represent significant GO terms, we selected random features of equivalent number per latent factor and carried out the ontology analysis. We repeated this process thrice and found that no significant term was associated for either the gene or the CpG features. We note that, from the list of top ranked features at different thresholds per latent factor, there are features that are shared by the latent factors. The number of these shared features increases, as the threshold decreases, from 99^th^ percentile to 25^th^ percentile. This confirms that higher the *W* co-efficient of a feature, higher is the contribution to the latent factor.

### CpG features in regulatory categories are enriched in distal enhancer locations

**Fig 4:**
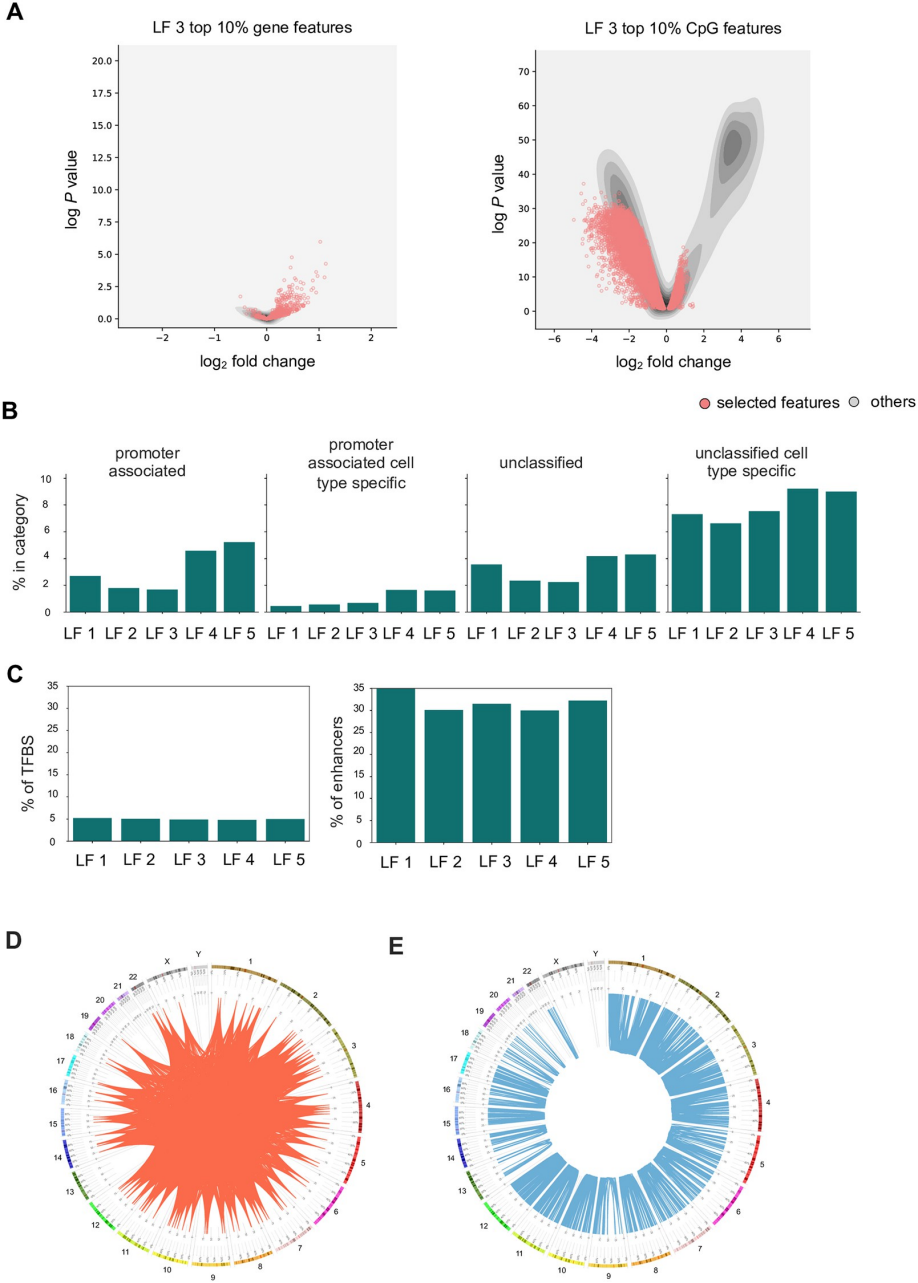
Characterization of features involved in correlations. **A)** Volcano plots showing the selected features for latent factor 3. The red dots represent the selected features which is plot against a kernel density estimate representation of all the gene/CpG features. **B)** The CpG sites were binned into regulatory categories of promoter associated and promoter associated cell type specific. **C)** For each CpG part of the selected features, the percentage of annotated enhancers and transcription factor binding sites is denoted by bar plots. **D)** The correlations are characterized in terms of the distance between the interacting partners, for the gene and CpG pairs derived from a latent factor analysis. The red ribbons on the CircOS plot show the connection between the interacting gene and CpG pair having a significant correlation. For this plot, top 5000 such significant correlations part of latent factor 3 were selected. **E)**. The correlations are characterized in terms of distance between interacting partners for m-QTL analysis. Here, the blue ribbons represent the significant correlating pairs (top 5000 randomly selected for m-QTL analysis of genes part of latent factor 3). The visualization emphasizes that the correlations obtained for latent factor analysis are distal (*trans*-acting) in nature.

We were interested in further dissecting the biological relevance of the identified features. First, we checked where the selected features fall in terms of log-fold change between DCM and controls. As an example, Fig 4A shows volcano plots with selected features for latent factor 3 marked in red on top of all measured features (mRNAs and CpGs, respectively) plot with density representation. Here, latent factor 3 features are representing mainly up-regulated genes in DCM and predominantly lower methylated CpGs in DCM. Next, we evaluated whether the CpG sites represent known enhancers, promoters or transcription factor binding sites as detailed in [43] (Fig 4B and 4C). We carried out a Fischer’s exact test to check whether the selected CpG sites (combined list for all 5 latent factors) were enriched for a particular regulatory category as compared to the rest. There is a 2.61-fold enrichment (*P* = 1E-06) of enhancers in the CpG sites part of latent factors, and an 8.22-fold enrichment (*P* = 1E-08) of promoters in the CpG sites that are part of the latent factors. We also counted the number of known transcription factor binding sites, but they are not significantly overrepresented in the chosen factors.

As described in the methods section, we also used the selected features to perform a correlation analysis between the selected gene expression and CpG features. We wanted to study the genomic proximity of the correlating gene and CpG pairs. The correlations derived from the latent factors mostly consist of *trans*-acting pairs, with an average of 94% of the correlating pairs not present on the same chromosome. This is in stark contrast with the associations derived from m-QTL analysis, which are *cis*-acting (Fig 4D and 4E).

### NMF features are enriched for positively correlated interacting partners not found using m-QTL analysis

**Fig 5:**
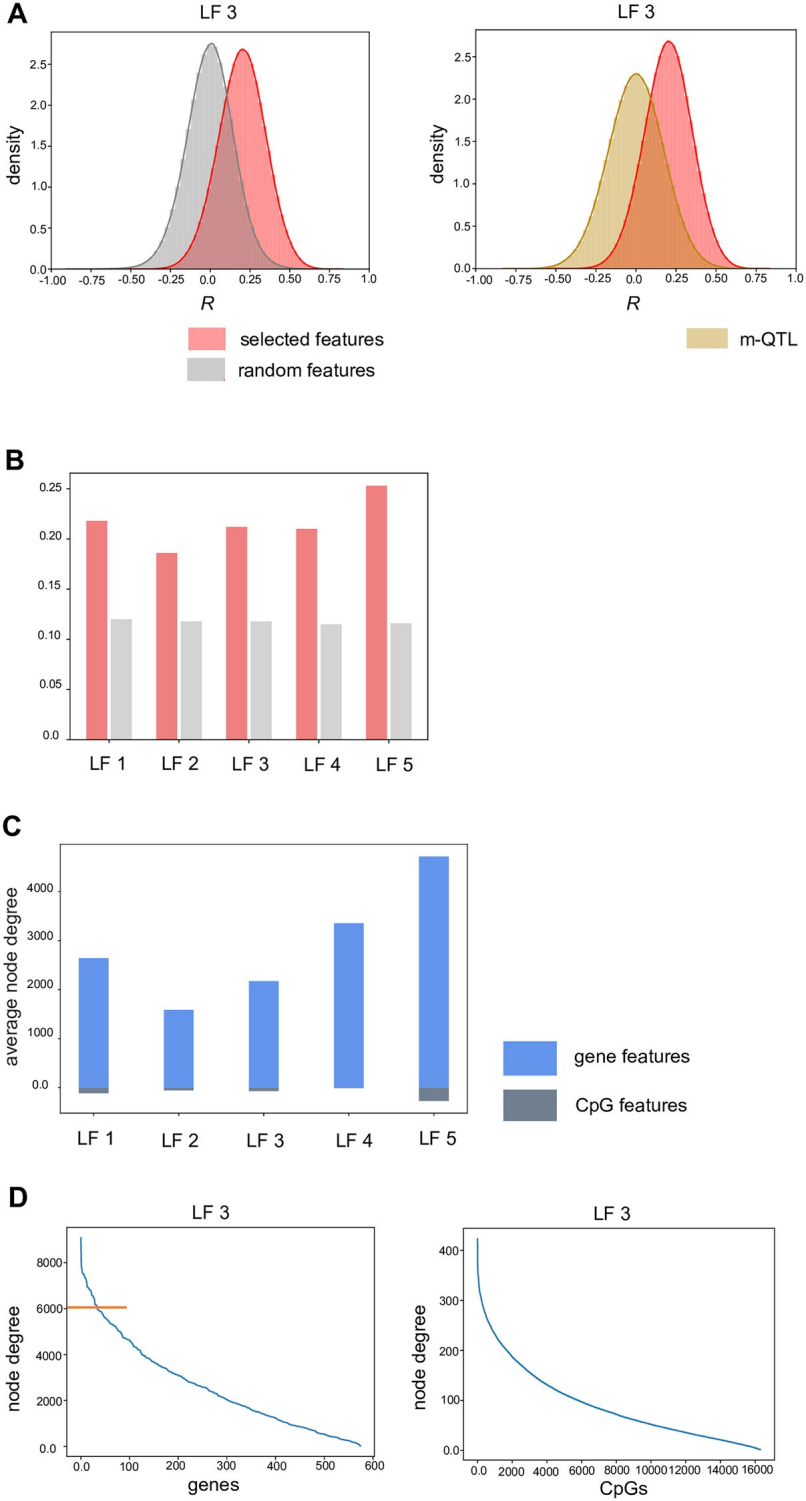
Distribution of the correlation coefficients for latent factor analysis and analysis of node degree of the resulting network. **A)** The figure represents the distribution of the correlation coefficients for the correlations analysis performed for selected gene and CpG features (for LF 3). This distribution is compared to a distribution obtained by random selection of gene and CpG features. It is also compared to the distribution obtained in the m-QTL analysis. **B)** The barplots represent the mean *R* for the latent factor analysis as compared to random background. **C)** Average node degree for gene and CpG features per latent factor is represented. **D)** The sorted node degree values for all gene and CpG features for latent factor 3 are shown. The orange line represents the 90^th^ percentile cut-off used for further analysis of high node-degree genes.

After selection of features per latent factor, their characterization and correlation analysis, we inspected the strength of the *R* values (S8 Fig) Top correlations are provided in the S2 Table. We then compared it against correlation between randomly selected features. The mean *R* of the selected features is higher than mean for randomly selected feature pairs. This is an evidence for the fact that the algorithm predominantly finds groups of positively co-regulated gene and CpG features in our datasets, which might reflect the underlying disease context. Hence, we asked if the found correlations could also be obtained using an m-QTL analysis, in which the selected gene features are associated with CpG features in their vicinity. We considered the selected gene features and CpG sites within 10 kilo bases of the transcriptional start site of each gene. Then, we performed an m-QTL analysis and retained the correlations which had a significant *P* value after FDR correction. We compared the correlations with the latent factor analysis correlations and found that on an average, 97% of the correlations are unique to latent factor analysis (Table 4). The distribution figures also show that the correlations from latent factor analysis are shifted towards positive correlations as compared to m-QTLs (Fig 5A and 5B, S5 Fig).

**Table 4:** Summary of total number of significant correlations in each analysis.

Latent factor	Number of significant correlations	Unique in latent factor analysis
LF 1	2082072	2005984 (96.34%)
LF 2	1929635	1884588 (97.66%)
LF 3	1488015	1465263 (98.47%)
LF 4	128467	126417 (98.40%)
LF 5	5045921	4946793 (98.03%)

The table shows the total number of correlations per latent factor and the number of correlations that are unique to a latent factor analysis as compared to the m-QTL analysis.

### Analysis of node degrees in correlation networks shows association of genes with several CpG sites

Networks were created from the correlations calculated for features per latent factor and the node degree was analyzed. Node degree is indicative of the connectivity of a feature to other features. Gene features have a much higher average node degree than CpG features, suggesting that a given gene has many interacting CpG partners. Fig 5C summarizes the average node degree for gene and CpG features and the S6 Fig depicts the magnitude of sorted node degrees for the networks derived for latent factors. As an example of the magnitude of node degree, Fig 5D shows the sorted node degree for latent factor 3 gene and CpG features.

**Fig 6:**
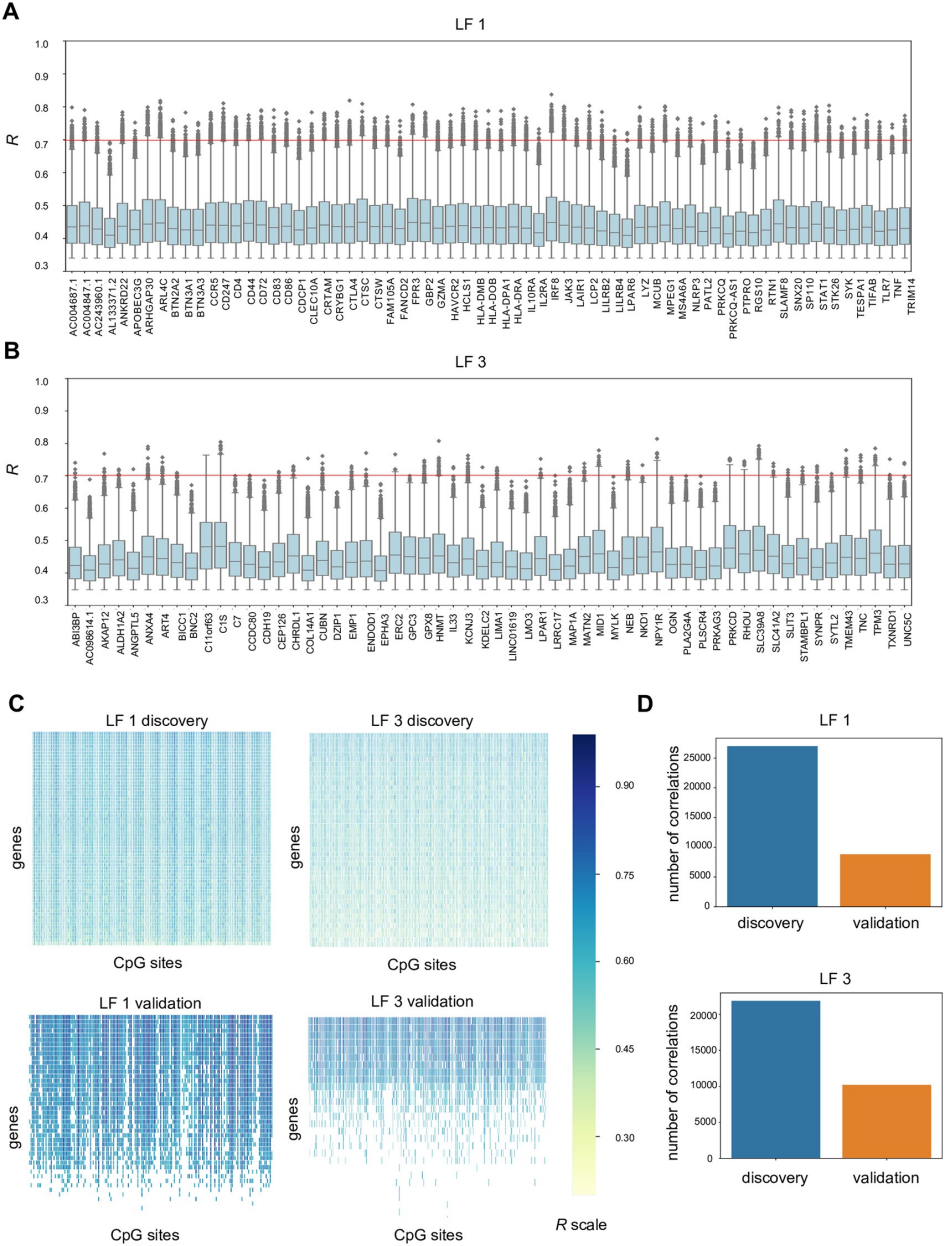
Distribution of correlation coefficients for the gene features within 90^th^ percentile of node degree for latent factor 1 and latent factor 3. **A)** Box plots represent the correlation coefficients of the gene features that fall into the 90^th^ percentile of node degree for latent factor 1. **B)** Similarly, boxplots for the features belonging to latent factor 3 are represented. **C)** The contingency matrix shows the top correlations (*R*>0.7) with the gene and CpG features for latent factor 1 and 3, along with the contingency matrix for the validation cohort. **D)** The bar-plots represent the number of correlating pairs part of the discovery and validation cohorts.

For further exploration of the correlation results, we looked at the gene features that have a node degree within the 90^th^ percentile. Boxplots summarizing the correlation coefficients of high node-degree genes give an idea of their distribution (Fig 6A and 6B and S7 Fig). While most of the *R* values are in the range of 0.35 to 0.45, several associations have *R* of 0.7 and above. For latent factor 1, these associations involve many genes related to immune system process, as described earlier. These include the genes *IRF8*, *CTLA-4*, *SLAMF8* and others, which are downregulated in DCM as compared to controls–or theoretically vice versa since the controls receive immunosuppressive medication after their heart transplantation. Hence, in an independent replication cohort with DCM cases and road side accident victims without prior medication with immunomodulators, highly correlating pairs (*R*>0.7 in discovery cohort) could be replicated in 28% (Fig 6C and 6D). For latent factor 2, a predominance of genes coding for zinc finger proteins could be seen, e.g. *ZNF25*, *ZNF326*, *ZNF56*; along with other genes involved in the cellular component of nucleus e.g. *NUDT21*. As described earlier, latent factor 2 relates to nucleic acid binding, and we see an overall downregulation of this process in DCM as compared to controls. High node degree genes of latent factor 4 were *ACO1* (aconitase 1), *DYM*, *ANK2*, *SLC25A12* and *TMEM246* which are upregulated in DCM, albeit not at a individually significant level. High node degree gene features for latent factor 5 include several different genes, but there is a predominance of genes related to cellular component of mitochondrion, like the *CYC1* and *NDUFV1*.

Interestingly, the average *W* coefficient of gene features is higher than average *W* coefficient for CpG features for each latent factor. Thus, even though gene features are fewer in number, their average contribution to the latent factor is higher compared to CpG features (Table 5). We checked if the average *W* coefficient of the genes with a high node degree is higher than the average *W* coefficient of all gene features. Indeed, the absolute value is higher for the genes with high node degree. We can thus conclude that these genes with high node degree interact with more CpG sites on average and also contribute most to the latent factor. A comparison of the average log fold-change of the selected gene features and the average log fold-change of the gene features of a high node degree reveals that the latter tends to be higher than the former.

**Table 5:** Summary of mean *W* co-efficient and mean log-fold change for high node degree genes.

Latent factor	*W* genes	*W* gene high node	*W* CpG s	Log-fold change	Log-fold change high-node
LF 1	0.46	0.47	0.43	-0.44	-1.31
LF 2	0.67	0.68	0.63	-0.01	0.07
LF 3	0.58	0.61	0.54	0.19	0.25
LF 4	0.89	0.90	0.88	0.10	0.13
LF 5	0.62	0.63	0.59	-0.09	-0.06

Table contains the average *W* coefficients for gene and CpG features and for gene features with high node degree. As can be seen from the table, the *W* coefficient for genes is higher than CpG features, even though the cumulative *W* is high for CpG sites. Also, the cumulative log fold-change for the selected features per latent factor is summarised in the table. The cumulative log fold-change for the gene features with high node degree is also provided.

### Differentially expressed genes belonging to latent factor 3 reveal a regulatory network of sarcomeric constituents

Since latent factor 3 represents the processes of sarcomere, myofibril and collagen containing extracellular matrix, it is of special interest in the context of DCM and therefore we carried out a deeper exploration into these associations. The top correlations involved the genes *C1S*, *HNMT*, *NPY1R*, *ANXA4*, *SLC39A8* and others. The list of gene features having a high node degree (within the 90^th^ percentile) comprises a total of 58 genes, for example, components of extracellular matrix (*COL14A1*, *CCDC80*, *ABI3BP*, *ANXA4*, *GPC3*, *OGN*), actin cytoskeleton (*NEB*, *TPM3*, *LIMA1*, *MYLK* and *EPHA3*) and the endoplasmic reticulum lumen (*TNC*, *TMEM43* and *GPC3*). Mean log-fold change for the 58 genes is 0.25 and the mean *W* is 0.61. Both these values are higher for the 58 genes than for the 576 genes selected for LF3. The boxplot in Fig 6 shows the distribution of correlation coefficient for the 58 genes. We selected the correlations *R*>0.7 and inspected the associations. The corresponding contingency matrix is shown in Fig 6C. Of these highly correlating pairs (LF3), as much as 45% could be validated in the independent validation cohort (Fig 6D).

**Fig 7:**
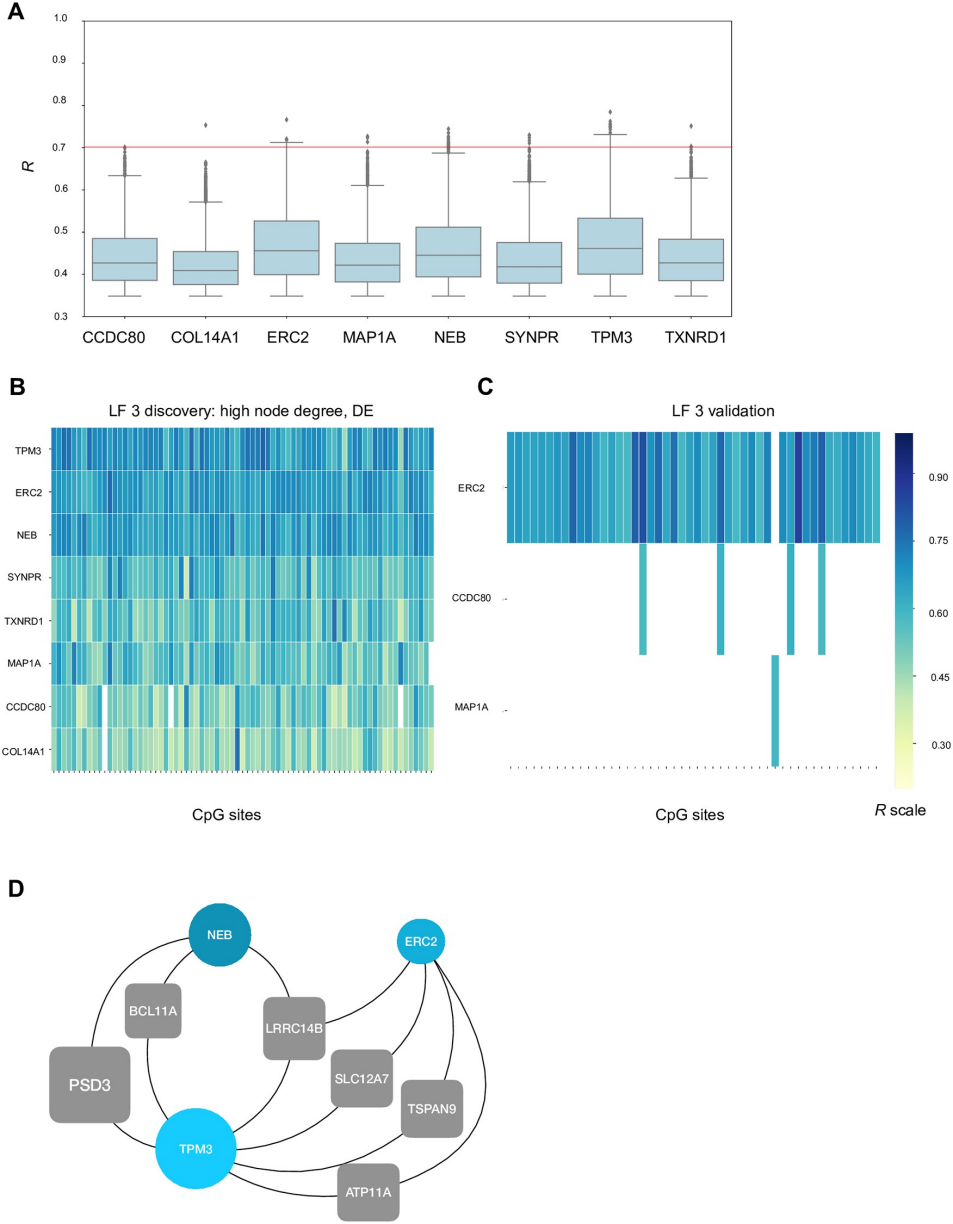
Top gene features for latent factor 3 with differential gene expression between DCM and controls. **A)** The boxplots depict the correlations for the genes that have a high node degree and are also significantly differentially expressed between DCM and controls. **B**) The contingency matrix depicts the correlations for high node degree and significant DCM association features in the discovery and validation cohorts. All CpG sites are not listed in the contingency matrix, refer to S10 Fig for the full list. **C)** For the *TPM3*, *NEB* and *ERC2* genes, we plot a network for the CpG sites shared between them. The blue nodes represent the genes and the grey boxes represent the CpG sites. The CpG sites are named by the genes that they are part of.

We further explored the associations of not only the genes with a high node degree, but also the nodes with significant differential expression between DCM and control, effectively choosing genes that show high interconnection and association with DCM. Consequently, gene features which have a high node degree (90^th^ percentile) and which are also significantly differentially expressed in DCM as compared to controls (*P*<0.05 FDR) were selected, which results in the following genes: *ERC2*, *CCDC80*, *NEB*, *TXNRD1*, *COL14A1*, *TPM3*, *SYNPR*, *MAP1A*. Boxplot in Fig 7A shows an overview of the distribution of correlation coefficients for the genes. We again set a threshold of 0.7 for *R* value and inspected the correlations, which totaled to 90. Fig 7B and 7C show the contingency matrices depicting strong correlations in which the said 8 genes are part of, both in discovery and validation cohorts. In the discovery cohort, out of 90 correlating pairs, 75 unique CpG sites on 60 genes are present. The CpG sites included in these associations are part of genes related to several processes like muscle tissue morphogenesis and contraction–*FGFR2*, *VEGFA*, *LRP5*, *MYBPC3* and ion-transport related genes like *ATP11A* and *SLC12A7*. From this network, the highest node degree involves the genes *NEB*, *TPM3* and *ERC2*. We further considered the associations of only these three genes with CpG sites that interact with at least two of these genes (node degree > = 2). An example of the resulting networks is given in Fig 7D. The CpG site on the *LRRC14B* gene interacts with the *TPM3* (*R* = 0.78, *P* = 3.84E-07), *NEB* (*R* = 0.74, *P* = 2.55E-06) and *ERC2* (*R* = 0.70, *P* = 1.49E-05) genes. Additionally, this network consists of CpG sites present on the genes *ATP11A*, *TSPAN9*, *SLC12A7*, *BCL11A* and *PSD3*.

**Fig 8:**
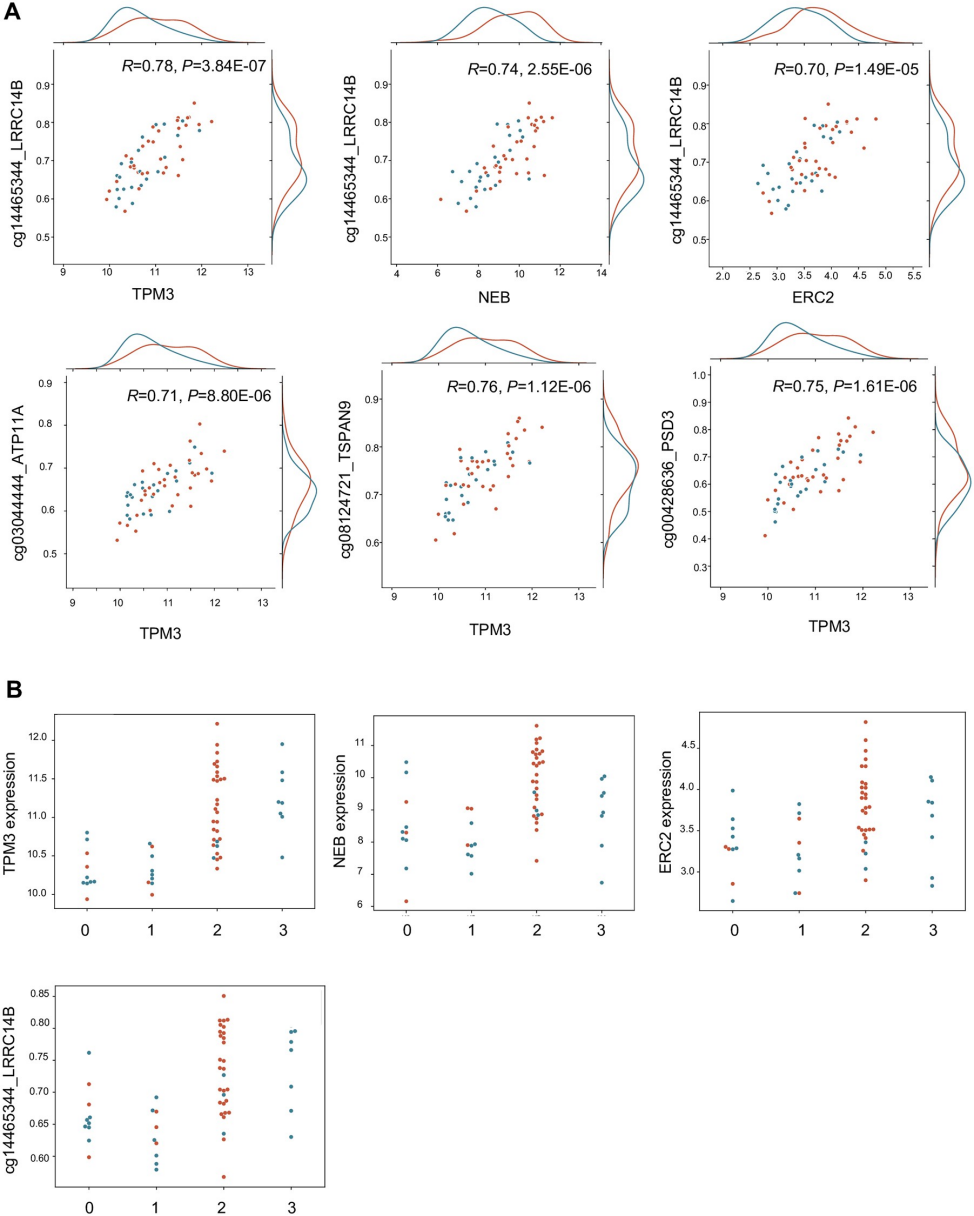
Scatterplots showing the association between key gene and CpG features. **A)** Scatterplots showing the correlation of *ERC2*, *NEB* and *TPM3* with the CpG site on *LRRC14B*. Additionally, it represents some of the highly correlating gene and CpG pairs involving high node-degree and differentially expressed genes. **B)** The expression of the genes and the CpG methylation is visualized by partitioning the samples into clusters as determined using the latent factor profile. The expression of the *NEB*, *TPM3* is particularly high in the cluster 2, which also has a high number of DCM samples.

For the gene and CpG features part of latent factor 3, we also carried out an ANOVA test to check whether the expressions of these genes are significantly different in the sample clustering performed with the latent factor profile *H* matrix. The gene expression across the clusters had a significant difference in expression, with *TPM3* (*P* = 6.23E-5), *NEB* (*P* = 7.05E-4) and *ERC2* (*P* = 1.12E-3). Scatterplots in Fig 8A depict the association of the three genes with the CpG site on *LRRC14B*. Swarmplot in Fig 8B depict the expression of the genes and the CpG site categorized as per clusters obtained using the *H* matrix.

## Discussion

NMF proved valuable for combining methylation and RNA-seq data from human tissue. The analysis presented in this paper serves as an example of how NMF can be used for the integration of bi- (or multi-) omics datasets and for patient stratification and discovery of co-regulated modules. Matrix factorization is an unsupervised approach and the only pre-filtering that was done on the data was to remove features that had a low mean expression and low variance between samples. NMF made it possible to approximate the high dimensional data into a lower number of factors and the resulting latent factor profile helped in clustering samples and identifying underlying biological information. K-means clustering could then be used on the reduced number of dimensions which mitigated the “curse of dimensionality” [44]. All latent factors are in turn related, as evidenced by hierarchical clustering of the 5 factors. Moreover, it was possible to group features from the two data modalities via the factors, and thus integration of RNA-seq and methylation data could be achieved.

At higher thresholds like 75^th^ percentiles, the number of common CpG features between the latent factors is high, and the subsequent GO term analysis reflects this fact. As we move to a lower threshold, we find that the selected features represent distinct pathways. Apart from representing distinct pathways, the selected features tend to have a positive and higher degree of correlation strength as compared to a random set of features, showing that the integrated gene and CpG features may indeed be co-regulated. Use of correlations to create networks is a common approach and has been used in gene co-expression studies [45, 46]. Further analysis of the generated correlations using network-based analysis helped us to discover the hub features and correlations between distal interacting partners.

Selecting top features representing each latent factor helped to reduce the computational burden to only few (example 576*16370 for latent factor 3) correlation calculations. Often, when performing a genome-wide association study (GWAS), correlations are calculated between all adjacent genomic features. For example, one study by our group [36] correlated methylation with adjacent splice-sites obtained from RNA-seq data. Selecting features through NMF could be seen as a way of reducing the number of total correlations to be performed. Thus, in this study we perform correlations similar to GWAS using features derived from NMF. The method of using NMF for matrix decomposition and use in a genome-wide association study has been performed before, where the latent factors are used as phenotypes for the GWAS. For example, in the study by [47], independent component analysis was used for finding latent factors, which then were used for association testing with SNPs. Also, the combination of GWAS and co-expression network analysis has been used to derive associations between data types [48, 49].

As far as multi-omics DCM studies are concerned, NMF has not been used as much as other traditional methods. The traditional methods usually test the significance of association of features to the disease individually and then find overlapping significantly differentially expressed features from different types of omics datasets [50]. While these methods are certainly beneficial, NMF provides an overview of the inter-relationship between all features, and how that maps to the samples as well, through latent factors, independent of the annotation level of the different features.

The analysis presented has potential limitations. In theory, the matrix factorization could be carried out at several different ranks to recover finer sources of variation in the data, but for the purpose of this study, we restricted the rank to 5 since the gain in explained variation is little after rank 5. Also, as shown in the S4C Fig, at rank 5, the extracted features strongly represent specific GO terms as compared to lower ranks. In the future, it would be worthwhile to explore more latent factors and find out what sources of small variation can be biologically meaningful. A known aspect of matrix factorization is that factorization will not always lead to the same solution. We tried to address this by performing three factorizations at rank 5 using different seeds. Each time, the five factors obtained the same biological processes. In this work, we concentrated on results obtained using the implementation available in NMF package. In the future, several implementations of NMF can be used and compared [51, 52]. An obvious shortcoming of the study is that the number of samples is not comparable to the number of samples used typically in GWAS studies [53, 54]. By combining the different biological layers and validating our results in a second cohort, we could reduce, however, the burden of false positives.

Despite these potential limitations, we could make useful observations with regards to the data, confirming already known as well as generation of novel knowledge. The latent factors represent known pathways/processes underlying the myocardium (immune system, nucleoplasm, extracellular-matrix and so on) and the features selected per factor help in understanding which gene and CpG features contribute to these pathways. We observe that biological processes are a concerted effect of several molecular players, and individual gene/CpG sites cannot be looked at in isolation. This was a top-down approach, where we described latent factors, pathways and then zoomed into individual components. Considering the cumulative log-fold change of the genes being part of the selected features we observed that the effect sizes per gene are below the top ranked candidates in traditional differential expression analysis. It is interesting to note that when selecting only the genes with high node degree, the mean log-fold change and mean *W* is higher than the one obtained using all selected genes.

Each latent factor warrants a detailed study, however, for this study we focused on latent factor 3, in order to have a more vertical probing of the data. For latent factor 3, GO term analysis from the list of selected features showed enrichment for myofibril and extracellular matrix. Known DCM markers like *NPPA* and *NPPB*, even if absent from this list, do have high coefficient on latent factor 3 as compared to other latent factors. From the correlation analysis performed for the selected list of features, we decided to perform further exploratory analysis for gene features with high node degree. We observed that many structural genes part of the sarcomere and extracellular matrix have strong correlations with CpG sites present on ion-transporters. From this list of genes, we then focused on genes with a high node degree which are also differentially expressed and discover a highly correlated group of features, depicted in the network in Fig 7D.

*NEB* is an important component of the thin filament and has been described in the context of heart failure and DCM before [55–57]. *TPM3*, a gene part of the tropomyosin family, is an actin binding protein and takes part in muscle contraction [58]. *TPM3* and *NEB* interactions have been described [59]. *ERC2*, part of the Rab interacting molecule (RIM) family of proteins, has not been found in the context of cardiomyopathies. However, it is known that these RIM proteins interact with voltage gated Ca(2+) channels [60]. We show that the three genes (*NEB*, *TPM3*, and *ERC2*) have a significant association to a CpG methylation site on the *LRRC14B* gene locus. To the best of our knowledge, these interactions have not been described earlier. *LRRC14B* is a member of the Leucine-rich repeat containing superfamily [61, 62], from which the *LRRC10* gene is well studied and has a role in DCM [63–65]. In fact, in our data, *TPM3*, *NEB* and *ERC2* also have significant correlations with a methylation site on *LRRC10* (with *R* > 0.5), but not as significant as *LRRC14B*. This network also shows us that there is a strong interaction between sarcomeric genes and the ion-transporter/membrane protein genes like *ATP11A*, *SLC12A7* and *TSPAN9*, on a transcriptional regulation level. Thus, in this work, we use a completely unsupervised method to derive correlations that may be biologically relevant and showcase the strongly interacting group of gene and CpG features.

## Conclusion

To summarize, our approach of joint matrix factorization of gene expression and methylation data achieved the primary purpose of an integrated analysis of the two data modalities via the latent factors. The latent factors helped in the condensation of the high dimensional data into smaller number of features which also represent distinct biological pathways. The correlation network analysis resulted in finding hub genes per latent factor and also groups of strongly interacting gene and CpG features. The results are beneficial for understanding the interplay between methylation and gene expression in the myocardium and may help to further the understanding of the disease.

## Supporting information

S1 Fig RNA-seq data statistics. **A**) The scatterplot shows the relationship between the total number of fragments per sample and the percentage of fragments uniquely assigned to a feature in the GTF file. **B**) After read-counting using featureCounts, normalization of gene expression is done in DESeq2 and the MA plot showing the mean of normalized counts and the log fold change is shown. **C**) A volcano plot shows the log-fold change and the P associated with the log-fold change. The red dots represent the genes that are significantly differentially expressed (log fold-change > 1.0, log *P* value > 0.43) between DCM and control. **D)** Principal components analysis of the RNA-seq data. **E)** Expression of *NPPA* gene in normalized read counts. F. Expression of *NPPB* gene in normalized read counts.
(TIF)

S2 Fig Methylation data statistics. **A)** Principal components analysis of the methylation data. **B)** Volcano plot showing the log-fold change of CpG methylation and the *P* associated with the fold change between DCM and control. Here the dots in red denote significantly differentially methylated sites between DCM and control (log fold-change > 3.0, log *P* value > 0.41).
(TIF)

S3 Fig Hierarchical clustering of RNA-seq and methylation data matrices. **A)** First 5 principal components from RNA-seq data matrix consisting of normalized read counts was used to create a sample-sample distance matrix, which is visualized as a clustered heatmap. **B)** Similarly, first 5 PCs from the methylation data matrix was used for creating a sample-sample distance matrix and was visualized as a clustered heatmap.
(TIF)

S4 Fig Hierarchical clustering of RNA-seq and methylation data matrices using top 1000 variable features. **A)** The hierarchical clustering of samples using RNA-seq data with the top 1000 most variable genes is shown in the heatmap. **B)** The top 1000 variables genes were also used for a *k-means* clustering at k = 4. **C)** Hierarchical clustering and **D)** *k-means* for methylation data matrix, using top 10000 features.
(TIF)

S5 Fig Rank optimization for matrix factorization. **A)** Explained variance calculated for rank 2:99. After rank 5, the explained variance reduces significantly. **B)** Hierarchical clustering of the latent factors derived from four models, from rank 2:4. C. GO terms associated with each latent factor at all ranks.
(TIF)

S6 Fig Distribution of the *W* matrix coefficients for features for each latent factor. The density plots show, for each latent factor the distribution of coefficients for gene and CpG features. The red bars denote the thresholds for top 1% (a), 5% (b), 10% (c), 25% (d) and 75% (e) of the features.
(TIF)

S7 Fig Distribution of correlation coefficients for all the latent factors. The correlation coefficient distribution per latent factor is shown in the plot (red) along with the correlations derived for random pairs of features (grey). The correlation coefficient distribution for the validation cohort (brown) and the m-QTL analysis (golden) is also shown.
(TIF)

S8 Fig Sorted absolute correlation coefficients (*R*) per latent factor. The line plots show the absolute value of correlation coefficient, of the correlation between feature pairs for each latent factor. The number of correlations (per 1000) are plot on the X-axis.
(TIF)

S9 Fig Node degree for gene and CpG features. The sorted absolute node degree for the gene and CpG features per latent factor are shown as line-plots.
(TIF)

S10 Fig Distribution of correlation coefficients for high node degree features for latent factor 2, 4 and 5. List of high node degree genes for latent factor 2, 4 and 5 are shown, with the boxplots depicting the range of the correlation coefficients.
(TIF)

S11 Fig *W* coefficients for features in the 90^th^ percentile for 5 latent factors. **A**) The boxplots represent the *W* coefficient for the features selected for each latent factor. For example, for latent factor 1, the features falling within 90^th^ percentile have high *W* coefficient for latent factor 1, but lower values for other latent factors. **B**) The boxplots for *W* coefficient for the high node degree features is shown.
(TIF)

S12 Fig Contingency matrix showing the high correlations for high node degree and differentially expressed genes part of latent factor 3. Gene and CpG interacting pairs and the strength of their correlations is shown as a contingency matrix.
(TIF)

S13 Fig Network showing the centrality of *NEB*, *TPM3* and *ERC2*. Network created using the top correlations (*R* > 0.70) for the genes that have high node degree as well as are differentially expressed between DCM and control. The network shows that the genes *NEB*, *TPM3* and *ERC2* are major hubs.
(TIF)

S1 Table More description of the cohort. Clinical parameters of the cohort are described.
(DOCX)

S2 Table Information on the validation cohort. The validation cohort is described in detail.
(DOCX)

S3 Table RNA-seq data statistics. Mean and variance of the gene expression data matrix. The count is the total number of features the values are reported for, for three variables–mean, standard deviation and variance. The mean and standard deviation of the same variables are then reported, along with the quantiles (min, 25%, 50% 75% and max).
(DOCX)

S4 Table Methylation data statistics. Mean and variance of the methylation data matrix. Just as for RNA-seq data matrix, the tables reports the total number of features, with statistics for three variables–mean, variance and standard deviation.
(DOCX)

S5 Table Information gain from clustering. Information gain from clustering at methylation and RNA-seq data matrices.
(DOCX)

S1 File NMF runs and GO term analysis of selected features. The excel file documents details of the performed NMF runs. NMF was performed once each. at rank 2, 3, and 4 and repeated thrice at rank 5. The overlap between common selected features at each threshold is also shown. In addition, GO terms related to the selected features are listed.
(XLSX)

S2 File Node degree of the features per latent factor. The node degree of the features selected per latent factor is documented in descending order. Also, top 50 correlations per latent factor are reported.
(XLSX)
