# Supplementary figures and images for: Multi-omics assessment of dilated cardiomyopathy using non-negative matrix factorization
Source: PLoS One. 2022 Aug 18;17(8):e0272093. doi: 10.1371/journal.pone.0272093 (PMC9387871; doi:10.1371/journal.pone.0272093)

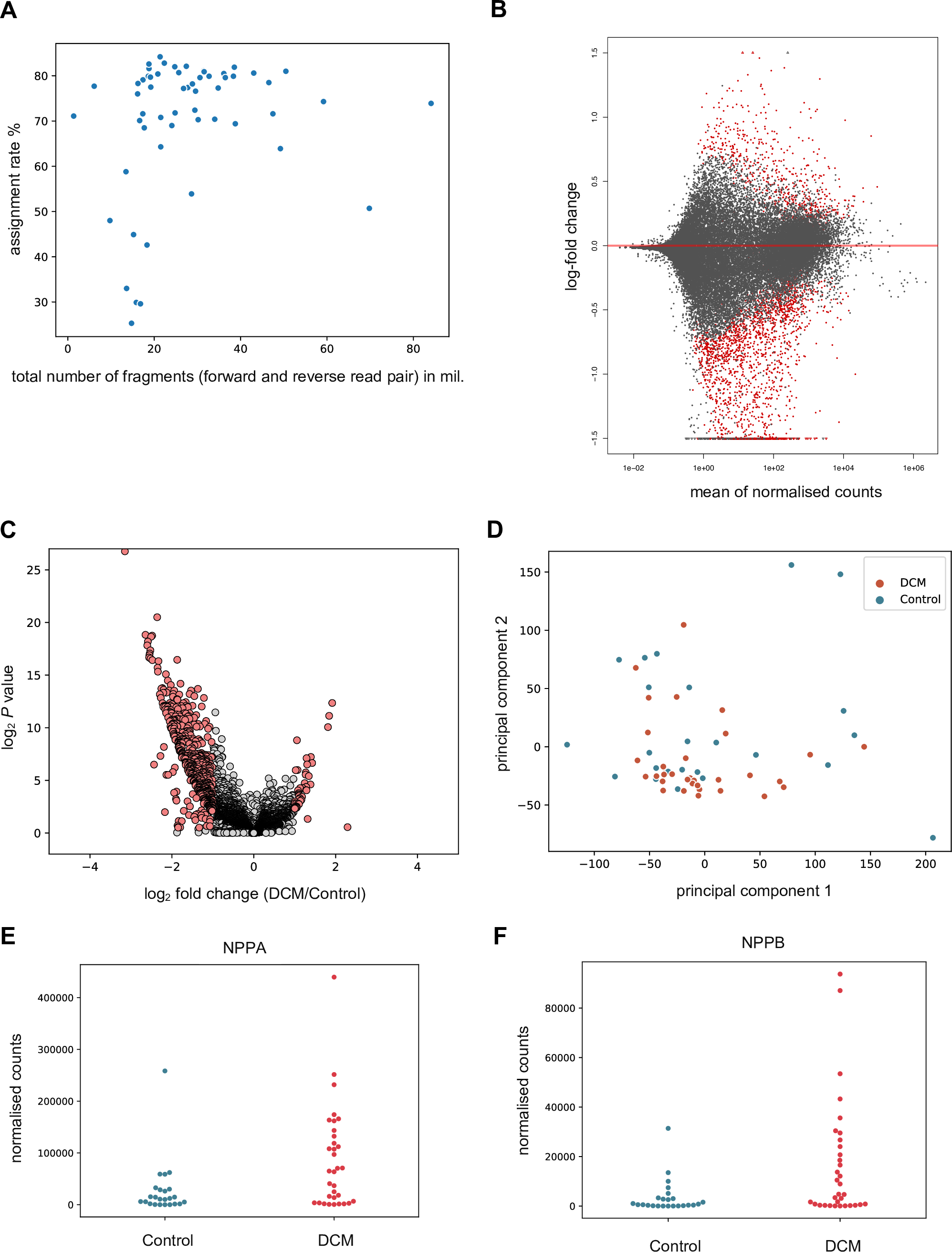

Supplement: S1 Fig — A) The scatterplot shows the relationship between the total number of fragments per sample and the percentage of fragments uniquely assigned to a feature in the GTF file. B) After read-counting using featureCounts, normalization of gene expression is done in DESeq2 and the MA plot showing the mean of normalized counts and the log fold change is shown. C) A volcano plot shows the log-fold change and the P associated with the log-fold change. The red dots represent the genes that are significantly differentially expressed (log fold-change > 1.0, log P value > 0.43) between DCM and control. D) Principal components analysis of the RNA-seq data. E) Expression of NPPA gene in normalized read counts. F. Expression of NPPB gene in normalized read counts. (TIF) [file pone.0272093.s001.tif]

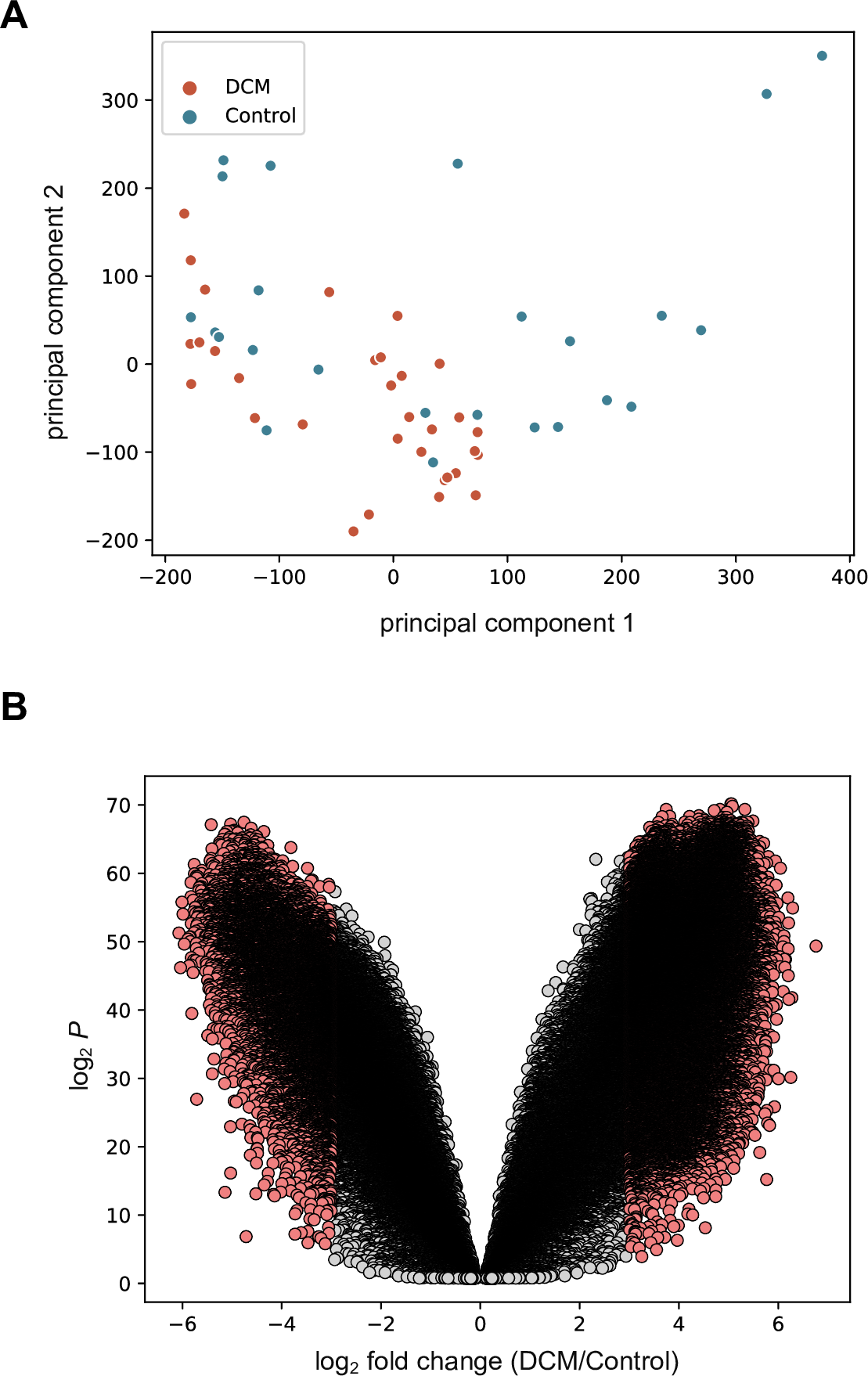

Supplement: S2 Fig — A) Principal components analysis of the methylation data. B) Volcano plot showing the log-fold change of CpG methylation and the P associated with the fold change between DCM and control. Here the dots in red denote significantly differentially methylated sites between DCM and control (log fold-change > 3.0, log P value > 0.41). (TIF) [file pone.0272093.s002.tif]

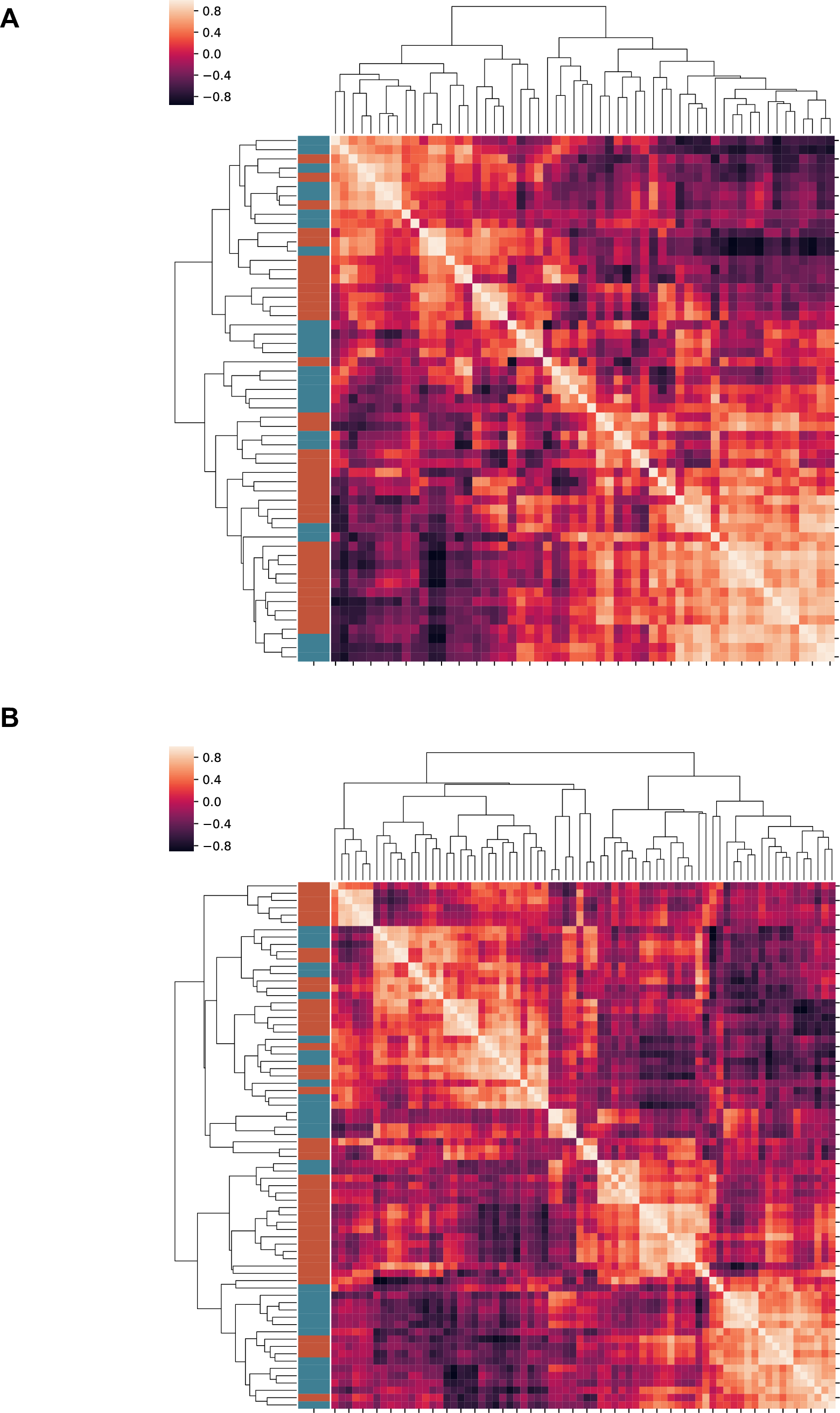

Supplement: S3 Fig — A) First 5 principal components from RNA-seq data matrix consisting of normalized read counts was used to create a sample-sample distance matrix, which is visualized as a clustered heatmap. B) Similarly, first 5 PCs from the methylation data matrix was used for creating a sample-sample distance matrix and was visualized as a clustered heatmap. (TIF) [file pone.0272093.s003.tif]

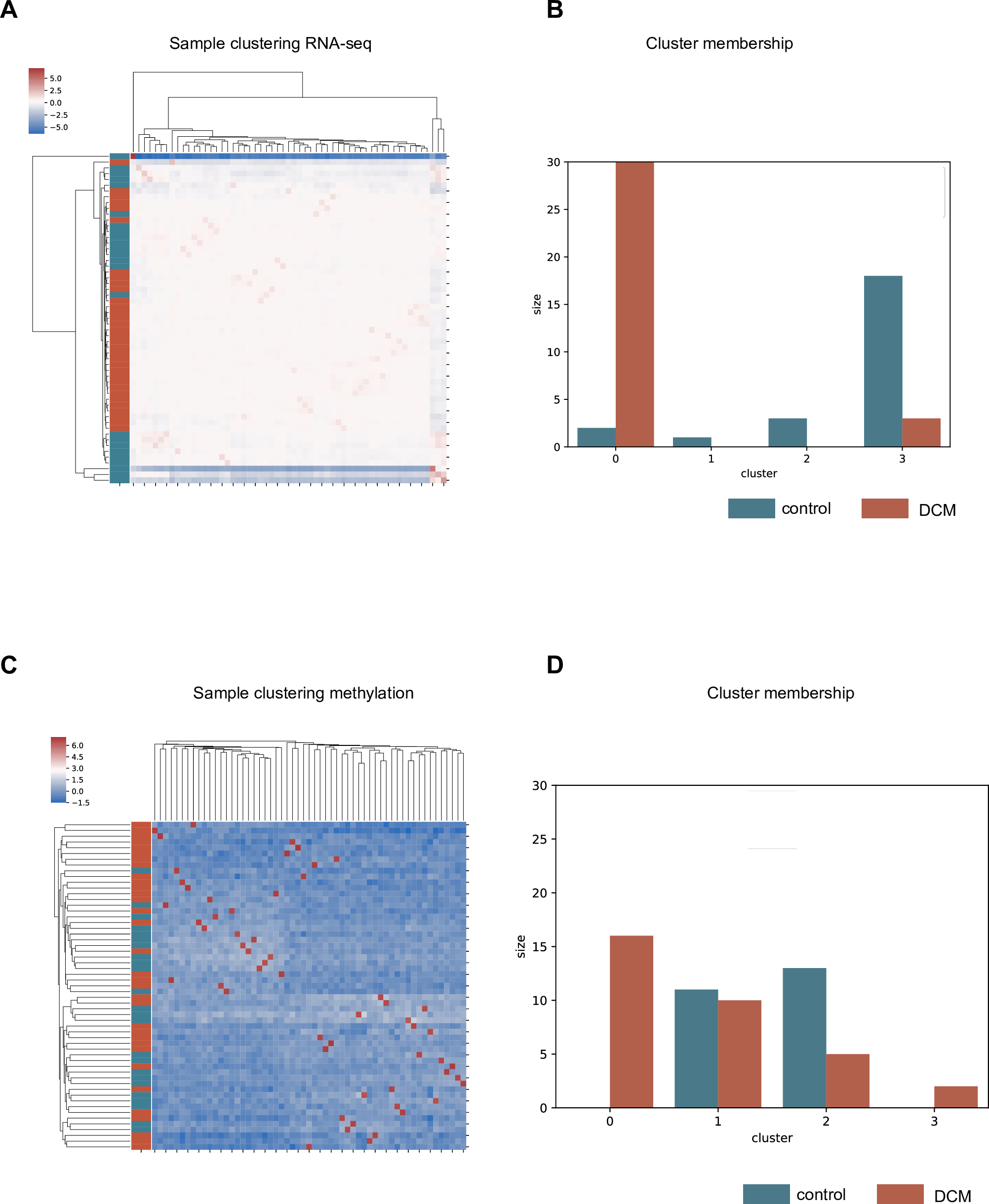

Supplement: S4 Fig — A) The hierarchical clustering of samples using RNA-seq data with the top 1000 most variable genes is shown in the heatmap. B) The top 1000 variables genes were also used for a k-means clustering at k = 4. C) Hierarchical clustering and D) k-means for methylation data matrix, using top 10000 features. (TIF) [file pone.0272093.s004.tif]

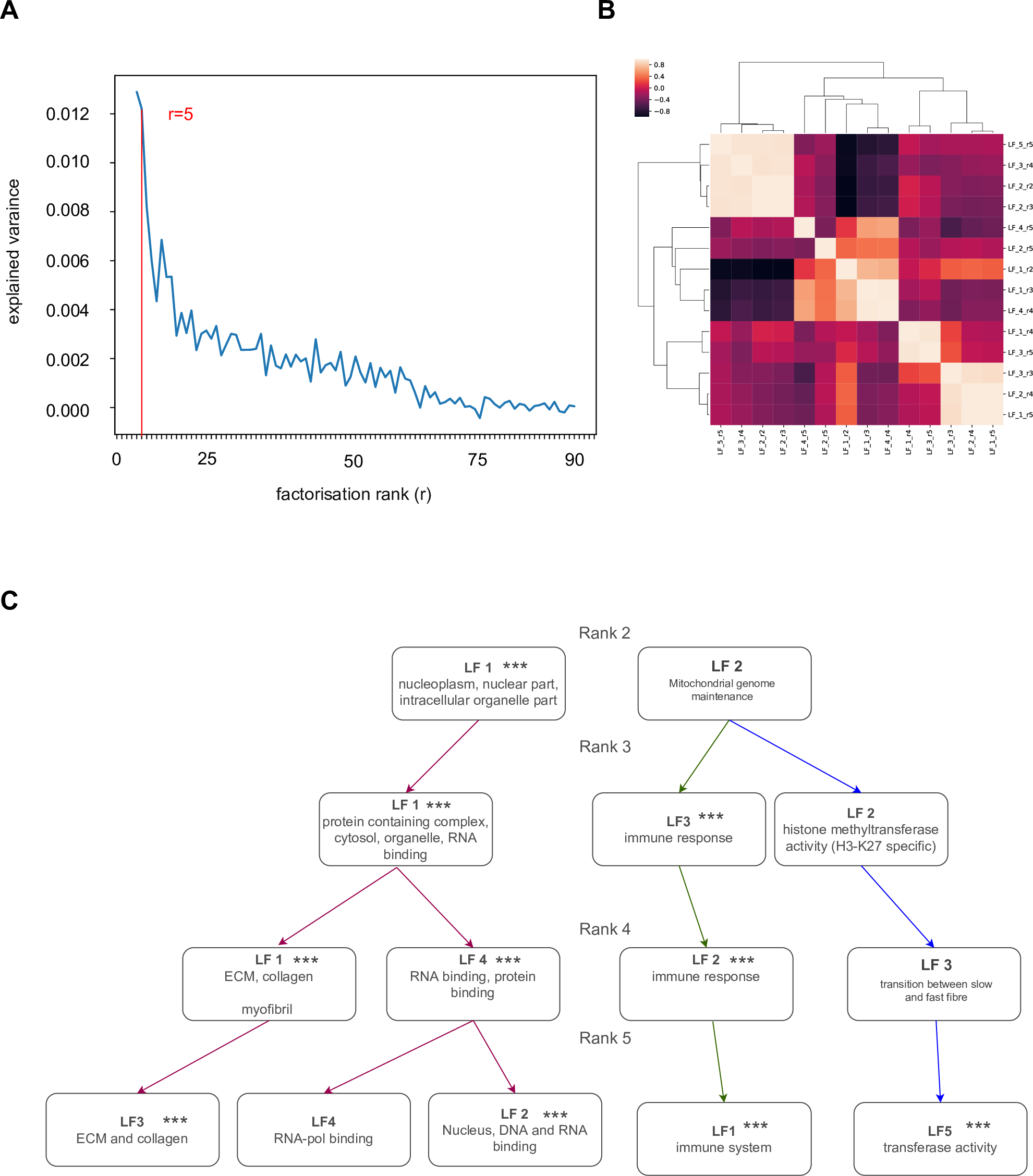

Supplement: S5 Fig — A) Explained variance calculated for rank 2:99. After rank 5, the explained variance reduces significantly. B) Hierarchical clustering of the latent factors derived from four models, from rank 2:4. C. GO terms associated with each latent factor at all ranks. (TIF) [file pone.0272093.s005.tif]

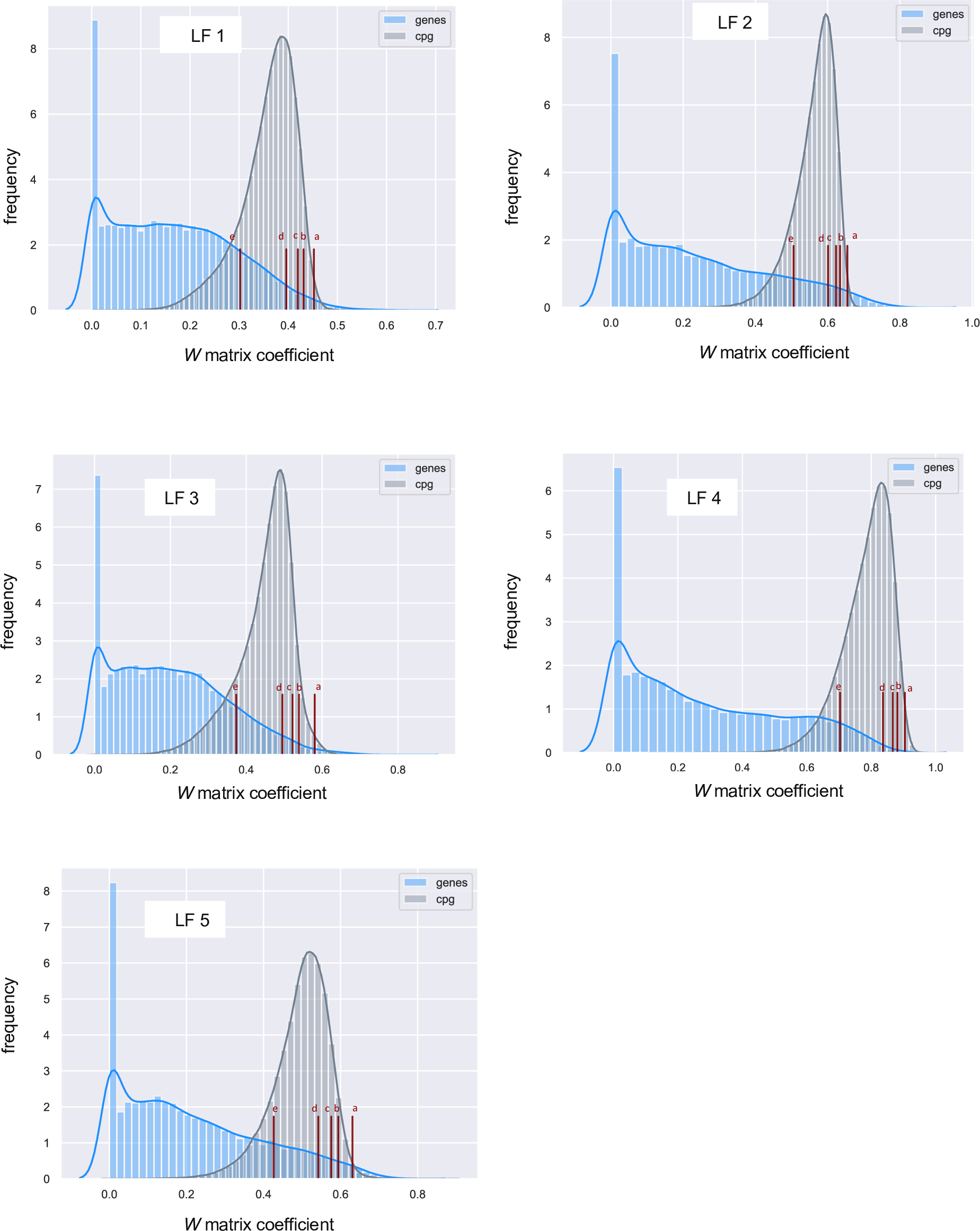

Supplement: S6 Fig — The density plots show, for each latent factor the distribution of coefficients for gene and CpG features. The red bars denote the thresholds for top 1% (a), 5% (b), 10% (c), 25% (d) and 75% (e) of the features. (TIF) [file pone.0272093.s006.tif]

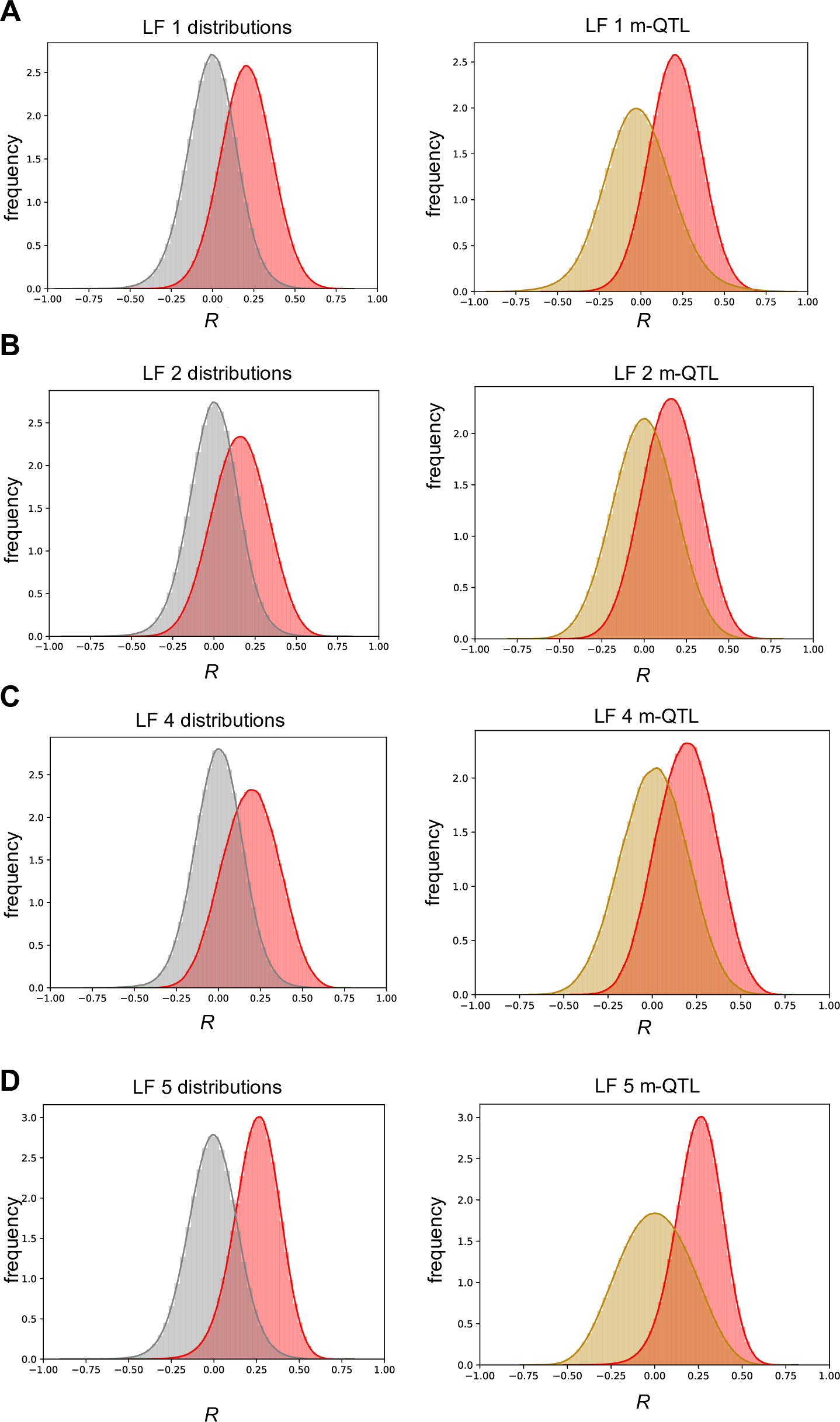

Supplement: S7 Fig — The correlation coefficient distribution per latent factor is shown in the plot (red) along with the correlations derived for random pairs of features (grey). The correlation coefficient distribution for the validation cohort (brown) and the m-QTL analysis (golden) is also shown. (TIF) [file pone.0272093.s007.tif]

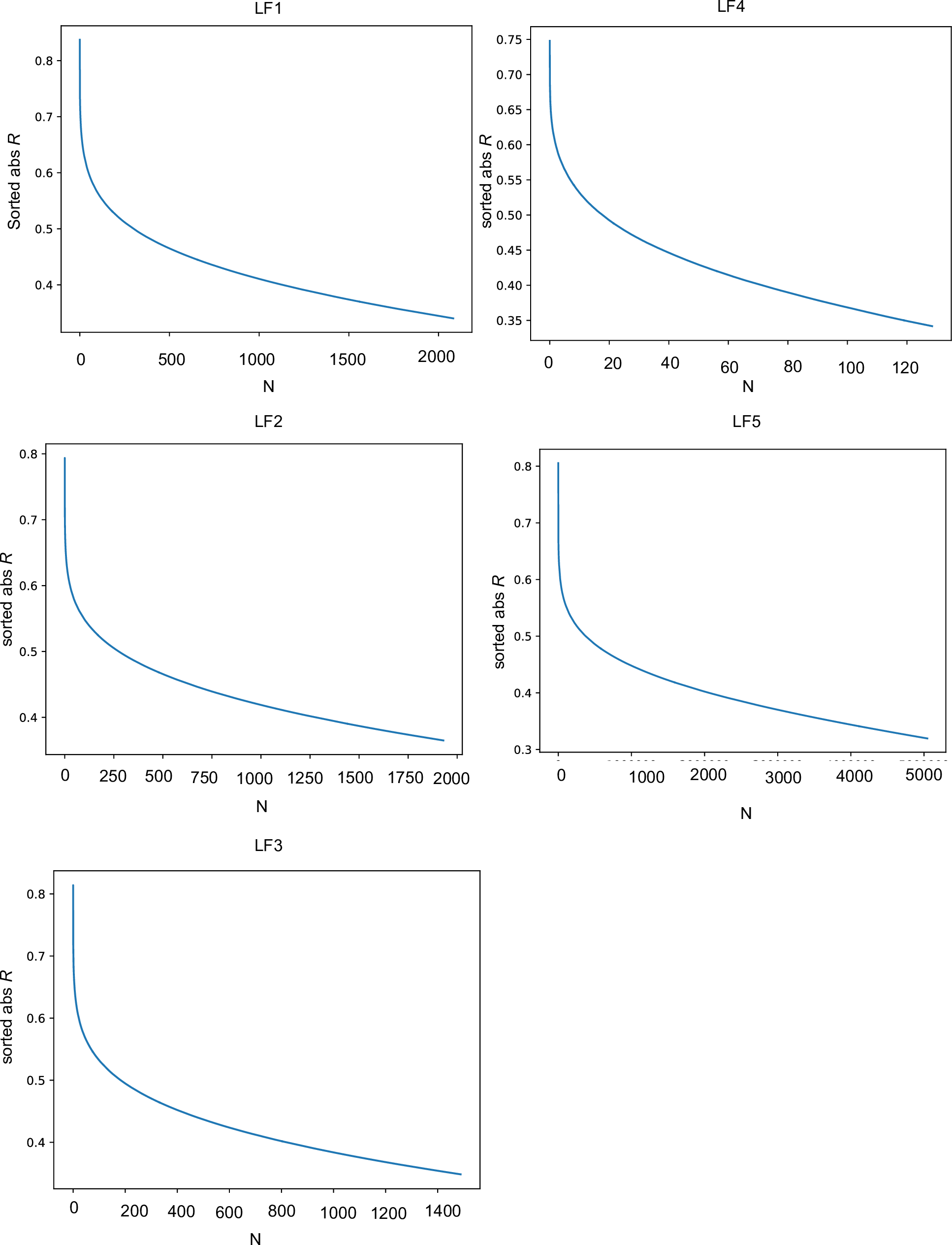

Supplement: S8 Fig — The line plots show the absolute value of correlation coefficient, of the correlation between feature pairs for each latent factor. The number of correlations (per 1000) are plot on the X-axis. (TIF) [file pone.0272093.s008.tif]

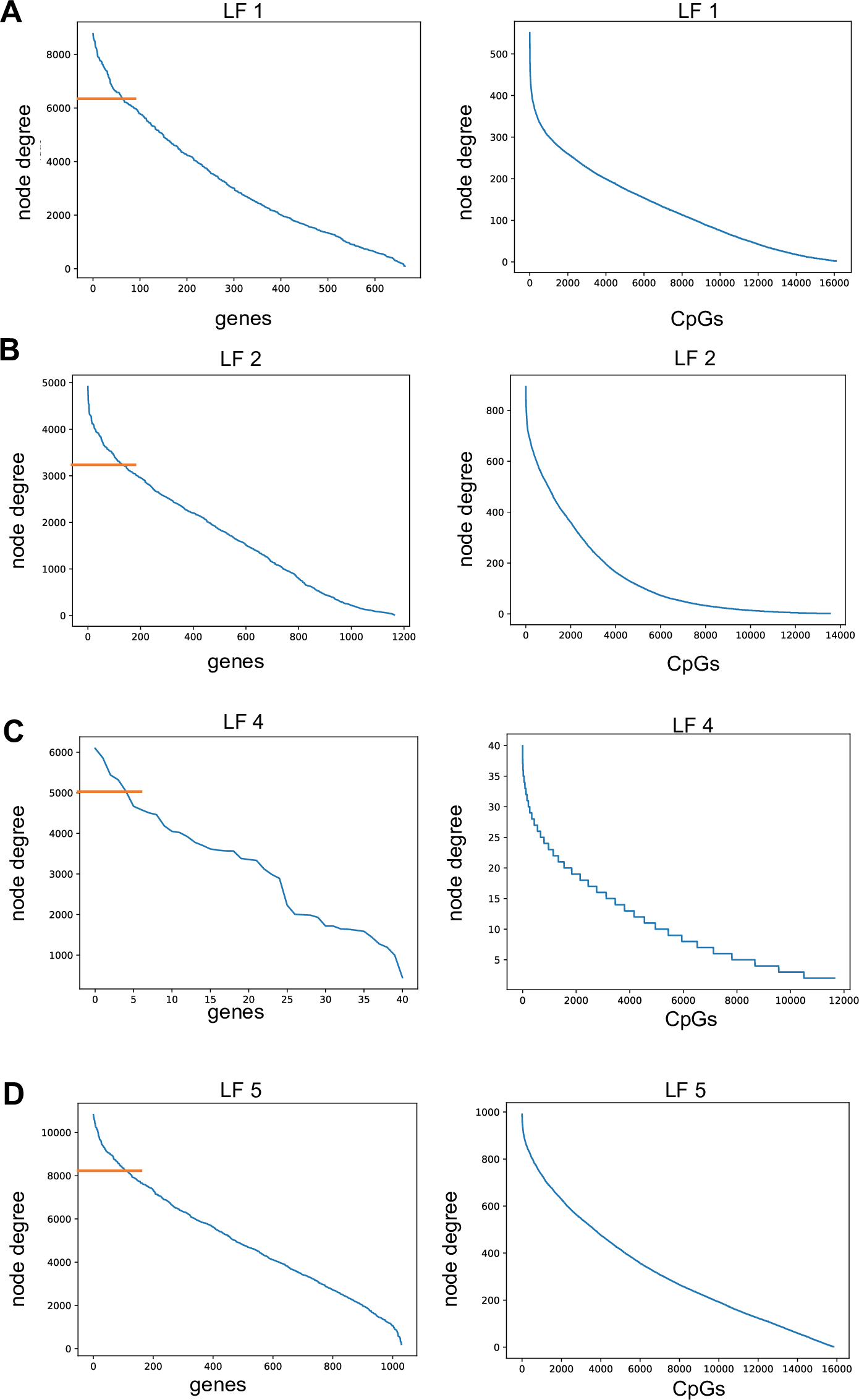

Supplement: S9 Fig — The sorted absolute node degree for the gene and CpG features per latent factor are shown as line-plots. (TIF) [file pone.0272093.s009.tif]

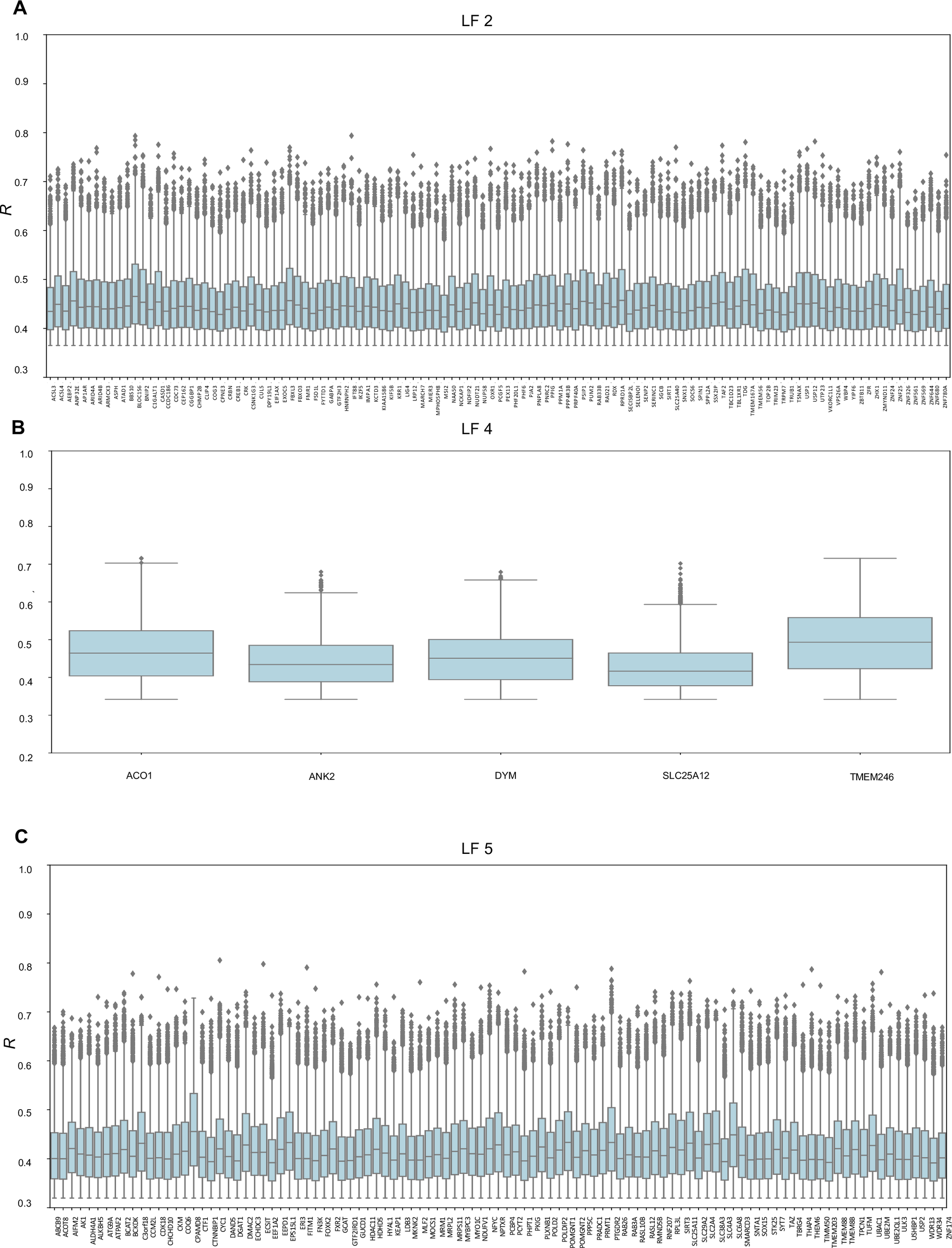

Supplement: S10 Fig — List of high node degree genes for latent factor 2, 4 and 5 are shown, with the boxplots depicting the range of the correlation coefficients. (TIF) [file pone.0272093.s010.tif]

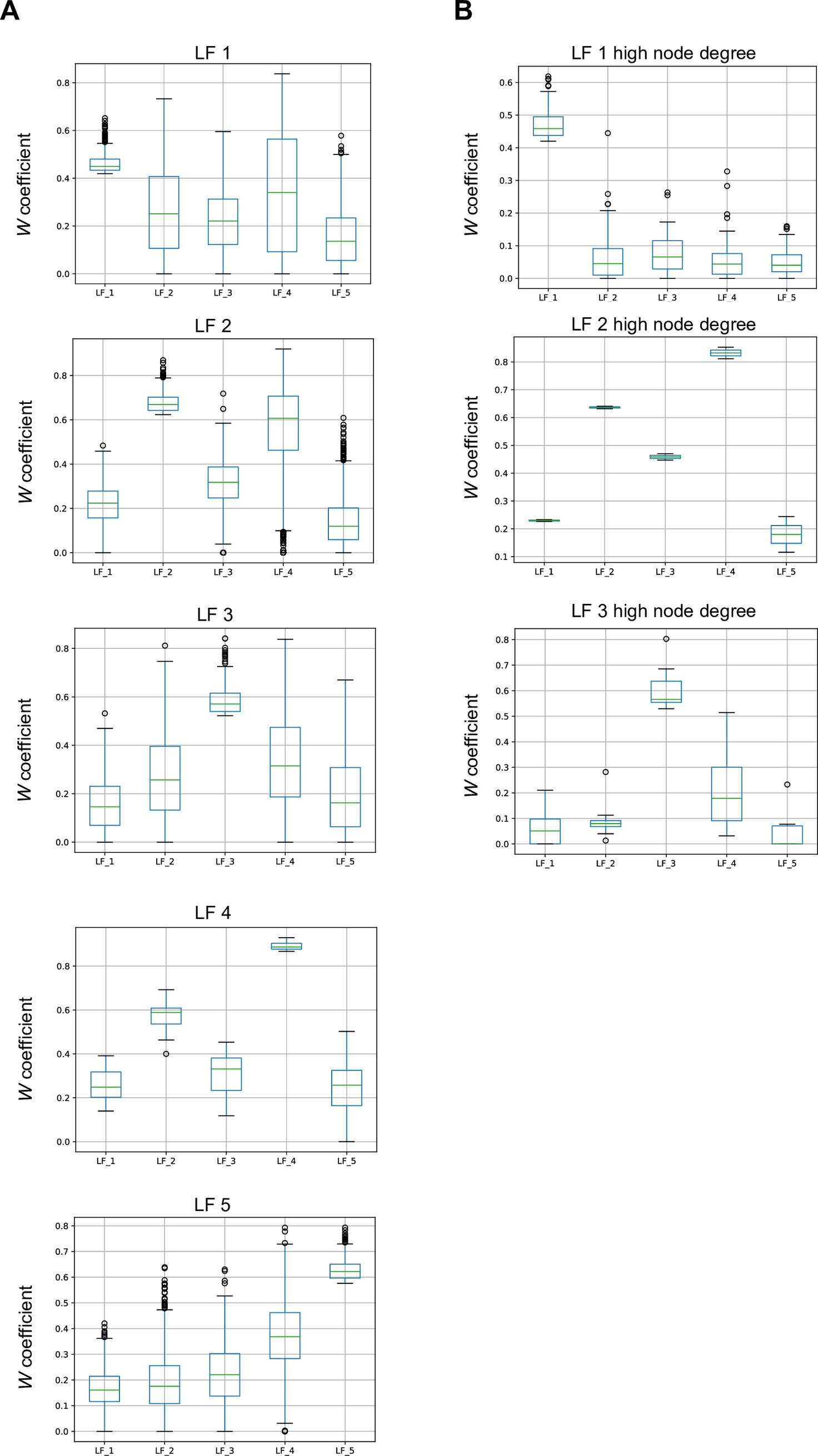

Supplement: S11 Fig — A) The boxplots represent the W coefficient for the features selected for each latent factor. For example, for latent factor 1, the features falling within 90th percentile have high W coefficient for latent factor 1, but lower values for other latent factors. B) The boxplots for W coefficient for the high node degree features is shown. (TIF) [file pone.0272093.s011.tif]

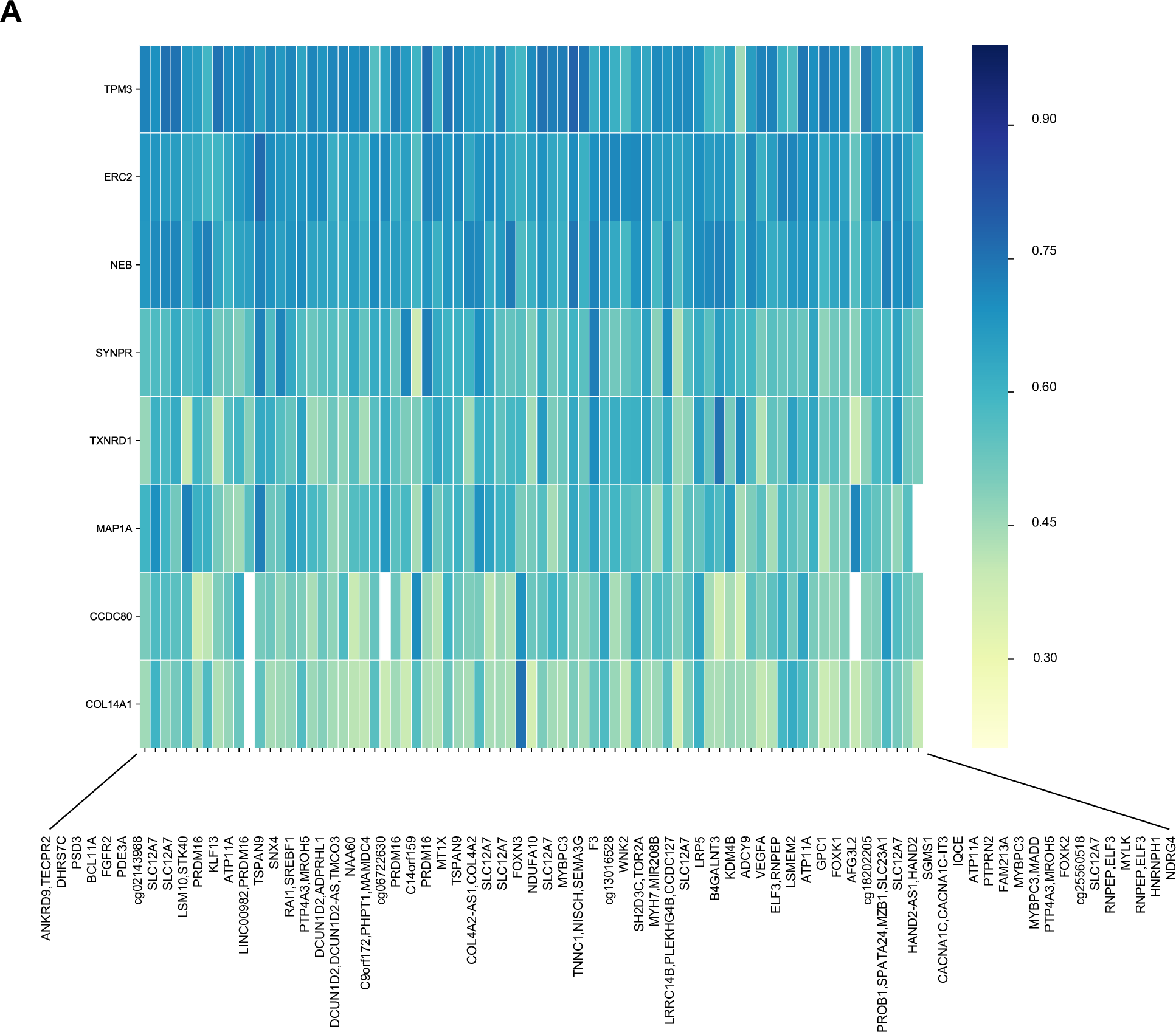

Supplement: S12 Fig — Gene and CpG interacting pairs and the strength of their correlations is shown as a contingency matrix. (TIF) [file pone.0272093.s012.tif]

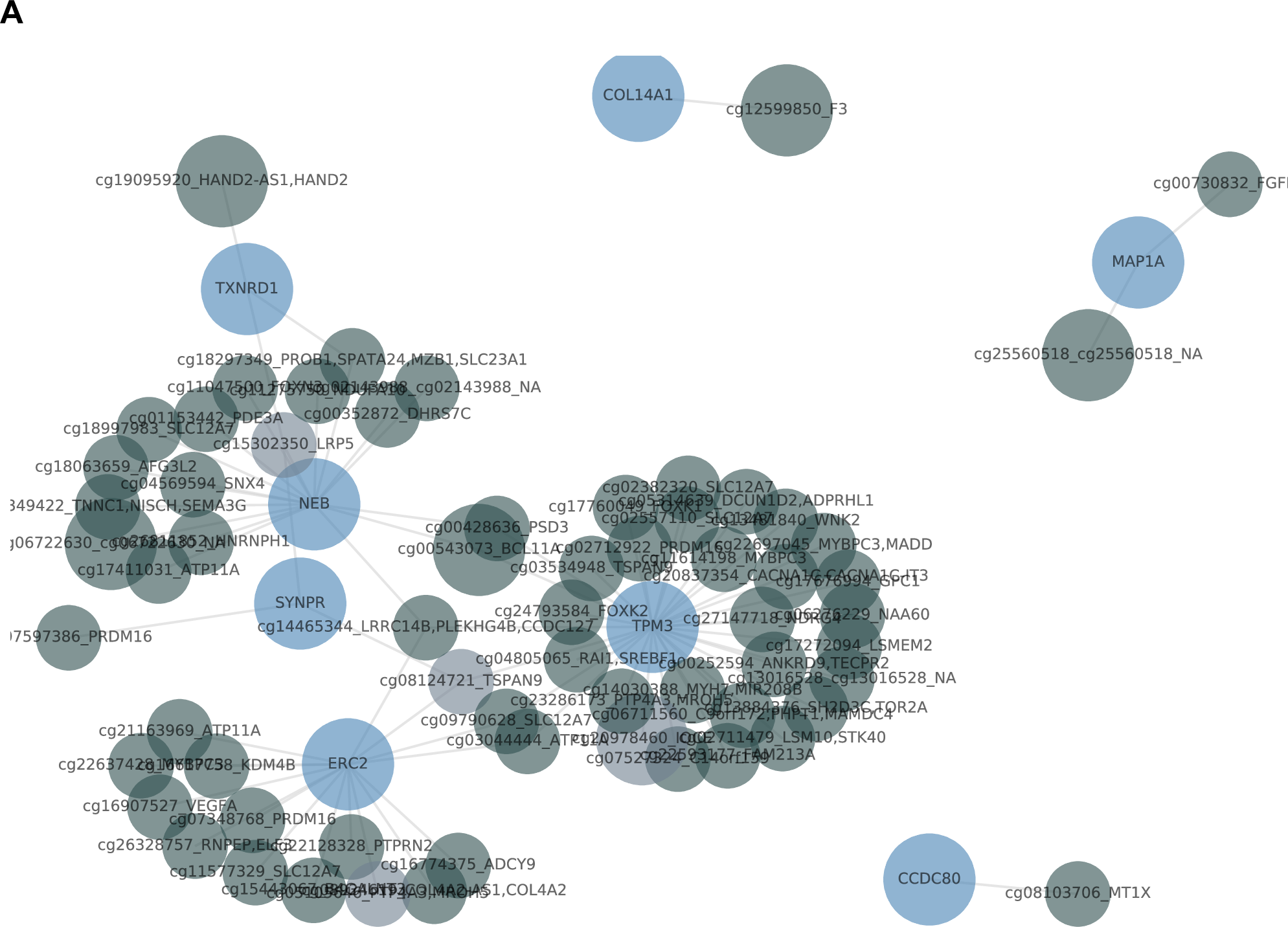

Supplement: S13 Fig — Network created using the top correlations (R > 0.70) for the genes that have high node degree as well as are differentially expressed between DCM and control. The network shows that the genes NEB, TPM3 and ERC2 are major hubs. (TIF) [file pone.0272093.s013.tif]
