# Supplementary material for: Multi-omics assessment of dilated cardiomyopathy using non-negative matrix factorization
Source: PLoS One. 2022 Aug 18;17(8):e0272093. doi: 10.1371/journal.pone.0272093 (PMC9387871; doi:10.1371/journal.pone.0272093)
Supplement: S1 Table — Clinical parameters of the cohort are described. (DOCX) [file pone.0272093.s014.docx]

**S1 Table. Summary of clinical parameters measured for the discovery cohort.**

| **Parameter** | **mean** | **std** | **min** | **max** |
| --- | --- | --- | --- | --- |
| Aldosteronantagonisten | 1.74 | 0.44 | 1.00 | 2.00 |
| LVEF-Ventriculography_ | 2.61 | 1.33 | 1.00 | 4.00 |
| Weight | 80.86 | 19.70 | 39.00 | 140.00 |
| ACE-H | 1.19 | 0.40 | 1.00 | 2.00 |
| BMI | 25.85 | 5.26 | 15.60 | 43.20 |
| ClinicID | 1239.84 | 174.28 | 1016.00 | 1498.00 |
| ASS | 1.53 | 0.50 | 1.00 | 2.00 |
| DIGITALis | 1.93 | 0.26 | 1.00 | 2.00 |
| Steroid | 1.84 | 0.37 | 1.00 | 2.00 |
| Age_at_Visit | 53.61 | 12.58 | 22.00 | 75.00 |
| ARB_AT1-Antagonisten_Sartane | 1.89 | 0.31 | 1.00 | 2.00 |
| Rhythm | 1.16 | 0.45 | 1.00 | 3.00 |
| BBL | 1.18 | 0.38 | 1.00 | 2.00 |
| height | 1.76 | 0.09 | 1.50 | 2.00 |
| NTprobnp | 743.88 | 1234.58 | 1e-21 | 6182.00 |
| Diuretikum | 1.58 | 0.50 | 1.00 | 2.00 |
| Statin_ | 1.25 | 0.43 | 1.00 | 2.00 |
| LBBB_RBBB | 3.09 | 1.14 | 1.00 | 6.00 |
| Gender | 1.21 | 0.41 | 1.00 | 2.00 |
| oral_anticoagulants | 1.91 | 0.29 | 1.00 | 2.00 |
