# Supplementary material for: Multi-omics assessment of dilated cardiomyopathy using non-negative matrix factorization
Source: PLoS One. 2022 Aug 18;17(8):e0272093. doi: 10.1371/journal.pone.0272093 (PMC9387871; doi:10.1371/journal.pone.0272093)
Supplement: S2 Table — The validation cohort is described in detail. (DOCX) [file pone.0272093.s015.docx]

**S2 Table. An overview of the validation cohort.**

|  | **Age at visit** | **Gender** |  |
| --- | --- | --- | --- |
| DCM | 57.11 | 5 females, 12 males | |
| controls | 49.12 | 5 females, 6 males | |
