# Supplementary material for: Multi-omics assessment of dilated cardiomyopathy using non-negative matrix factorization
Source: PLoS One. 2022 Aug 18;17(8):e0272093. doi: 10.1371/journal.pone.0272093 (PMC9387871; doi:10.1371/journal.pone.0272093)
Supplement: S3 Table — Mean and variance of the gene expression data matrix. The count is the total number of features the values are reported for, for three variables–mean, standard deviation and variance. The mean and standard deviation of the same variables are then reported, along with the quantiles (min, 25%, 50% 75% and max). (DOCX) [file pone.0272093.s016.docx]

**S3 Table. RNA-seq expression data basic statistics.**

|  |  |  | **mean** | **Std.Dev** | **variance** |
| --- | --- | --- | --- | --- | --- |
|  |  | count | 58302 | 58302 | 58302 |
|  |  | mean | 1.38 | 0.15 | 0.08 |
|  |  | std | 4.14 | 0.23 | 0.87 |
|  | Before filtering | min | -2.40 | 0.00 | 0.00 |
|  |  | 25% | -2.06 | 0.00 | 0.000007 |
|  |  | 50% | 0.00 | 0.05 | 0.0031 |
|  |  | 75% | 3.87 | 0.27 | 0.073 |
|  |  | max | 21.06 | 7.69 | 59.16 |

|  |  | count | 24026 | 24026 | 24026 |
| --- | --- | --- | --- | --- | --- |
|  |  | mean | 5.34 | 0.34 | 0.18 |
|  |  | std | 3.70 | 0.25 | 1.33 |
|  | After filtering | min | -2.33 | 0.0022 | 0.000005 |
|  |  | 25% | 1.97 | 0.226 | 0.051 |
|  |  | 50% | 5.59 | 0.30 | 0.091 |
|  |  | 75% | 8.44 | 0.40 | 0.163 |
|  |  | max | 21.06 | 7.69 | 59.16 |
