# Supplementary material for: Multi-omics assessment of dilated cardiomyopathy using non-negative matrix factorization
Source: PLoS One. 2022 Aug 18;17(8):e0272093. doi: 10.1371/journal.pone.0272093 (PMC9387871; doi:10.1371/journal.pone.0272093)
Supplement: S4 Table — Mean and variance of the methylation data matrix. Just as for RNA-seq data matrix, the tables reports the total number of features, with statistics for three variables–mean, variance and standard deviation. (DOCX) [file pone.0272093.s017.docx]

**S4 Table. Methylation data basic statistics.**

|  | **mean** | **Std.Dev** | **variance** |
| --- | --- | --- | --- |
|  | 394247 | 394247 | 394247 |
|  | 0.44 | 0.03 | 0.00 |
|  | 0.31 | 0.02 | 0.00 |
|  | 0.01 | 0.00 | 8e-6 |
| Before filtering | 0.10 | 0.01 | 2.81E-4 |
|  | 0.47 | 0.03 | 9.65E-4 |
|  | 0.75 | 0.04 | 2.35E-3 |
|  | 0.98 | 0.30 | 9.58E-2 |
|  |  |  |  |
|  |  |  |  |
|  | 145411 | 145411 | 145411 |
|  | 0.65 | 0.05 | 3.15E-3 |
|  | 0.12 | 0.01 | 3.58E-3 |
| After filtering | 0.40 | 0.03 | 9.65E-4 |
|  | 0.55 | 0.03 | 1.56E-3 |
|  | 0.66 | 0.04 | 2.41E-3 |
|  | 0.76 | 0.06 | 3.76E-3 |
|  | 0.96 | 0.30 | 9.58E-2 |
