# Supplementary material for: Multi-omics assessment of dilated cardiomyopathy using non-negative matrix factorization
Source: PLoS One. 2022 Aug 18;17(8):e0272093. doi: 10.1371/journal.pone.0272093 (PMC9387871; doi:10.1371/journal.pone.0272093)
Supplement: S5 Table — Information gain from clustering at methylation and RNA-seq data matrices. (DOCX) [file pone.0272093.s018.docx]

**S5 Table. Information gain.**

| **Data type** | ***k*=4** | ***k*=5** | ***k*=6** | ***k=*7** |
| --- | --- | --- | --- | --- |
| Latent factor | 0.43 | 0.42 | 0.50 | 0.41 |
| Methylation data | 0.34 | 0.36 | 0.19 | 0.28 |
| mRNA | 0.05 | 0.07 | 0.10 | 0.10 |
